# Expediting Lithium Electrochemistry via a Bilayer for High-Rate Lithium Metal Batteries

**DOI:** 10.1007/s40820-026-02146-3

**Published:** 2026-03-19

**Authors:** Dongjoo Park, Dong-Wan Kim

**Affiliations:** School of Civil, Environmental, and Architectural Engineering, Korea University, Seoul, 02841 Republic of Korea

**Keywords:** Lithium metal batteries, Lithium dendrites, Intermetallic lithium alloying, Electrospinning, High rate

## Abstract

The intrinsic characteristics of the Li metal anode, particularly its ultra-high specific capacity (3860 mAh g^−1^) and low redox potential (−3.04 V vs. SHE), theoretically make it ideal for high-rate charge/discharge operations. However, the high Li self-diffusion barrier causes uncontrolled plating/stripping dynamics and severe volume fluctuations, hindering stable performance at elevated current densities. In this study, we introduced an artificial solid-electrolyte interphase (ASEI) engineered with a bilayer that transcends conventional planar deposition, facilitating Li nucleation and growth along three-dimensional electronic percolation pathways. This spatially distributed, lateral plating morphology significantly reduced charge-transfer resistance, suppressed dendrite formation, and mitigated cell degradation under high charging currents. Consequently, the ASEI-enabled Li metal electrode maintained low overpotentials at an areal capacity of 10 mAh cm^−2^ and a current density of 20 mA cm^−2^ for over 300 h, while demonstrating outstanding rate capability and long-term cyclability in LiFePO_4_(LFP)‖Li and LiNi_0.8_Co_0.1_Mn_0.1_O_2_ (NCM811)‖Li full cells. By elucidating these intrinsic anode behaviors, our findings establish a fundamental design strategy for high-rate performance, potentially advancing the commercialization of Li metal batteries.

**Supplementary Information:**

The online version contains supplementary material available at 10.1007/s40820-026-02146-3.

## Highlights

- The bilayer combines an electron‐conductive framework and an ion‐conductive layer that facilitates Li⁺ transport, effectively separating charge pathways for enhanced electrochemical stability.
- Porous fibers with uniformly dispersed Ag nanoparticles provide nucleation sites for uniform Li plating and a 3D network that suppresses dendrite growth.
- The BL–Li anode shows a low overpotential of ≈120 mV at 20 mA cm⁻² and 10 mAh cm⁻², sustaining smooth Li deposition and stripping for over 300 h under high‐rate cycling.

**Supplementary Information:**

The online version contains supplementary material available at 10.1007/s40820-026-02146-3.

## Graphical abstract

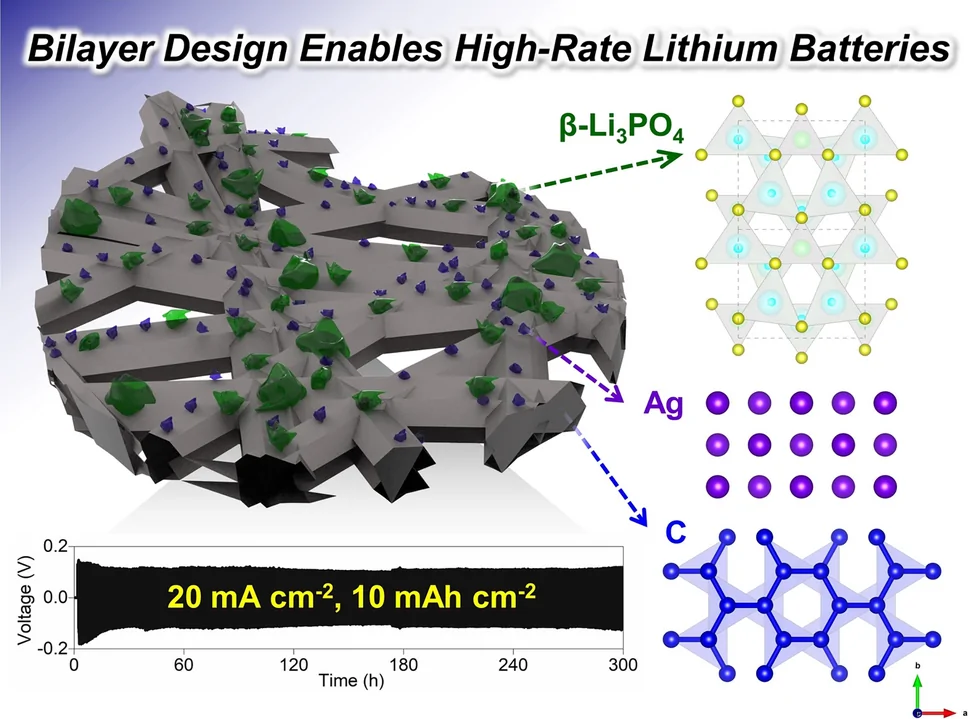

## Introduction

Li metal’s intrinsically low atomic weight and density and a high ionization tendency yield exceptional gravimetric/volumetric capacity (3860 mAh g^−1^, 2046 Ah L^−1^) and energy density (approximately 2600 Wh kg^−1^, 8000 Wh L^−1^) [1], making Li metal batteries (LMBs) a promising alternative to commercial Li-ion batteries, particularly for portable devices and electric vehicles. Li metal anodes offer intrinsically low ionic diffusion resistance as Li is directly electrodeposited onto the metal surface [2]. The interfacial reduction reaction shortens the charge-transfer diffusion path, lowers overpotential, and enables rapid charging [3]. However, the high nucleation overpotential of Li metal anodes renders microscopic surface imperfections susceptible to intense local electric fields [4]. These concentrated fields drive rapid ion reduction and plating at defect sites, promoting protrusion growth that accelerates dendritic formation via a positive-feedback loop [3, 4]. In addition, the SEI layer, formed at the low reduction potential of Li metal, repeatedly self-repairs during Li plating/stripping, continuously consuming electrolyte [3, 4]. Consequently, the electrode impedance increases, triggering short circuits and compromising Coulombic efficiency [3, 4]. To address these challenges, several promising strategies have been developed to reduce localized electric‐field concentrations at the Li interface and precisely modulate Li‐metal morphology [5–18]. Representative studies have demonstrated that lithium metal stability can be improved by employing protective composite layers fabricated via simplified processes [19].

An engineered artificial solid-electrolyte interphase (ASEI) at the Li/electrolyte interface mitigates localized electric-field intensification at the Li anode, suppressing dendritic growth and enhancing battery stability [11, 12]. Ag has a face-centered cubic structure similar to Li, with low lattice constant and surface diffusion barrier, promoting smooth Li electrodeposition [20]. However, controlling the agglomeration of high surface energy Ag nanoparticles while ensuring stable physicochemical bonding remains challenging. Electrospinning helps to fabricate an electronically conductive three-dimensional scaffold to ensure continuous electronic percolation path throughout the Li electrodeposited region [21]. Moreover, as a three-dimensional current collector, it increases the current distribution area for the Li metal electrode, reducing local current density and suppressing dendrite formation [22]. Ag nanoparticles uniformly anchored to the scaffold surface serve as homogeneous nucleation sites, lowering overpotentials via multistep Li–Ag alloying reactions and decreasing overall cell impedance [20, 23]. However, ASEIs focusing solely on electronic conductivity can permit electron migration through the scaffold and catalyze electrolyte reduction and decomposition, potentially exacerbating parasitic Li metal reactions [24]. Li_3_PO_4_, with negligible electronic conductivity and high Li-ion conductivity, reduces ion transport resistance at the electrolyte–Li interface [25, 26]. Its relatively high thermal decomposition temperature can also contribute to the thermal stability of battery [25].

In this study, a freestanding bilayer model without a bonding process is proposed via electrospinning. This work extends conventional electrospun architectures by enabling the formation of functionally differentiated bilayer structures. In a number of previous reports, bilayer (BL) models fabricated by casting or deposition involved the use of binders, which has been associated with reduced electrical conductivity [27–29]. The lithium surface passivation caused by the polymer can increase the low ion diffusion resistance of lithium metal and hinder the possibility of rapid charge/discharge. The porous morphology permits facile electrolyte infiltration without increasing lithium-ion diffusion resistance, and, since lithium can plate uniformly across the fiber surfaces, the deposited metal forms a 3D network that suppresses dendrite growth and dead-lithium formation. On a pure Li surface, the diffusion barrier of Li ad atoms is low enough such that small bumps or irregularities can be flattened to some extent. However, at high charging currents or uneven interfaces, the deposition rate can proceed much faster than the diffusion rate [30]. In such cases, dendrites formation occurs because of insufficient time to planarize the Li tip [4]. Abundant Ag nanoparticles in the bilayer drastically lowered the nucleation overpotential, promoting uniform Li reduction and deposition across the entire anode surface [31]. By maximizing the distribution of active sites and moderating local deposition rates, this approach yields smooth, rounded plating [32]. Abundance electron transport pathways equalize local current density, preventing local current concentrations and reducing dendrite formation and cell degradation reactions at high charge currents [30]. Rather than plating Li onto a conventional planar substrate, Li infiltrates the fiber network to form a three-dimensional matrix. During stripping, Li undergoes smooth electronic reionization and desorption, leaving no dead Li behind. By harnessing the intrinsic low ion diffusion resistance and short diffusion pathways of Li metal, this design enables rapid charge–discharge performance. The symmetric BL–Li cell exhibits a low overpotential of 118 mV at 20 mA cm^−2^ and 10 mAh cm^−2^ after 300 h. When paired with LFP or NCM811 cathodes, the battery maintains excellent rate performance and cycling stability, even under high loading and with a thin lithium anode. At a cathode loading of 8 mg cm^−2^, the LFP cell retains 80% of its initial capacity over 150 cycles, while the NCM811 cell at 10 mg cm^−2^ maintains 90% capacity retention over 160 cycles, both at 0.5  C. Moreover, when using a 30-μm-thick lithium metal anode, the BL-Li full cell records twice higher capacity with NCM811and extended cycle life with LFP compared to a bare-Li full cell.

## Experimental Details

### Materials

LiFePO_4_ (MTI Korea) and Li[Ni_0.8_Co_0.1_Mn_0.1_]O_2_ (NCM811, L&F Co.) served as the cathode materials. Li_3_PO_4_, AgNO_3_ polyacrylonitrile (PAN, Mw ≈150,000), polystyrene (PS, Mw ≈192,000), DMF, and DCM were obtained from Sigma-Aldrich. The electrolyte was formulated as 1 M LiTFSI in a 50:50 v/v mixture of DME/DOL with 1 wt% LiNO₃. Cathode slurries were prepared by mixing the active material with Kynar FLEX® 2801–00 (Arkema) and Super P (Alfa Aesar) in an 8:1:1 weight ratio using NMP (TCI). Celgard® 2400 monolayer membranes were used as separators in all cell assemblies.

### Preparation for Bilayer

The C-layer precursor was prepared by dissolving 0.648 g AgNO_3_ and 0.82 g PAN in 6 mL DMF and mixing for 20 min in a Thinky AR-100. This solution was then electrospun onto aluminum foil at 0.5 mL h^−1^ under 21 kV, with a 15 cm tip-to-collector distance and 500 rpm rotation for 2 h. Next, the L-layer solution—made by dissolving 0.6 g Li_3_PO_4_ and 4 g PS in 6 mL DMF + 6 mL DCM and stirring for 6 h—was electrospun directly atop the C-layer at 1.2 mL h^−1^, 21 kV, a 10 cm gap, and 500 rpm for 30 min to form the bilayer.

Finally, the assembled bilayer was annealed by ramping at 2 °C min^−1^ up to 300 °C, holding for 2 h, then continuing at 2 °C min^−1^ to 850 °C, and maintaining that temperature for 6 h.

### Materials Characteristics

Surface morphology and layer thickness were assessed with a Hitachi SU70 field-emission scanning electron microscope (SEM), a COXEM CX-200 SEM, and a JEOL JEM2100F high-resolution transmission electron microscope (TEM). Crystalline phases were identified by X-ray diffraction (XRD) using a Rigaku MiniFlex 600 (Cu K*α*). Bulk resistivity was measured on an Otsuka MCPD-3000 low-resistance meter, and surface elemental/chemical states were probed by X-ray photoelectron spectroscopy (XPS, Thermo Fisher K*α* +). Finally, cryogenic FIB (Aquilos) paired with cryo-TEM (Glacios at 200 kV and Krios G4 at 300 kV) was employed to study lithium dendrite formation.

### Electrochemical Measurement

CR2032 coin cells were employed throughout. Galvanostatic charge–discharge curves for Li‖Li symmetric, Li‖Cu asymmetric, and full-cell setups were recorded on battery cyclers (WonATech WBCS 3000; NEWARE CT-4008 T; MACCOR). The cathode slurries were prepared using a Thinky AR-100 planetary mixer. The slurry was mixed at 2000 rpm for 20 min and subsequently cast onto a 20-μm-thick aluminum current collector. The coated electrodes were dried overnight in an oven at 90 °C. The areal loading of LFP and NCM811 electrodes was approximately 2 mg cm^−2^. For high-loading electrodes, the active material, conductive additive, and binder were mixed in a weight ratio of 90:5:5. High-loading tests were performed with areal loadings of 8 mg cm^−2^ for LFP and 11 mg cm^−2^ for NCM811. Each was filled with 80 µL of electrolyte. The bilayer was applied by placing it directly onto the lithium metal in the lithium symmetric cell configuration, whereas in the Li–Cu asymmetric cell configuration used to investigate lithium deposition behavior, the bilayer was placed onto the Cu substrate. Lithium-ion conductivity (*σ*, S cm^−1^), Li⁺ transference number (t_Li⁺_), electrochemical impedance spectra (EIS), and Tafel plots were obtained using an IVIUM-n-STAT analyzer (IVIUM Technologies). Ionic conductivity was then determined via Eq. 1:

1 $$\sigma \, = l/ \, R_{b} A$$

where the thickness is (*l*, cm), the bulk resistance (*R*_*b*_ (Ω)), and the contact area (*A*, cm^2^). The Li transference number was calculated using Eq. 2:

2 $$t_{Li + } = I_{{\mathrm{s}}} \left( {\Delta V - I_{0} R_{0} } \right) \, /I_{0} \left( {\Delta V - I_{{\mathrm{s}}} R_{{\mathrm{s}}} } \right)$$

where the initial (*I*₀) and steady-state (*I*ₛ) currents, along with the initial (*R*₀) and steady-state interfacial resistances (*R*ₛ), were measured by EIS using a 1-mV perturbation over the 100 kHz–1 Hz range. In situ Raman spectra were acquired with a Cora 5001 probe (Anton Paar) coupled to the IVIUM-n-STAT analyzer. The LFP cathode loading was 1.2–1.5 mg cm^−2^, and the NCM811 loading was 2.3–2.6 mg cm^−2^.

The Li^+^ diffusion coefficient (*D*_Li+_) can be estimated from CV using the Randles–Sevcik Eq. 3:

3 $$I_{{{\mathrm{peak}}}} = 2.69 \times 10^{5} n^{1.5} AD_{{\text{Li + }}}^{0.5} C_{{\text{Li + }}} v^{0.5}$$

Here, *I*_peak_ represents the peak current, *v* is the scan rate, *D*_Li+_ denotes the diffusion coefficient of Li^+^, and *n*, *A*, and *C*_Li_^+^ refer to the number of electron equivalents per reactive species, electrode area, and lithium-ion concentration in the electrolyte, respectively.

### COMSOL Simulation

COMSOL Multiphysics was employed to simulate the electrodeposition behavior using a three‐species Nernst–Planck interfacial framework. A rectangular electrolyte domain (10 × 10 μm^2^) filled with 1 M LiTFSI in a DOL/DME mixture served as the model. The initial Li⁺ concentration was fixed at 1 M. Ionic conductivities were set to 7.68 × 10^−5^ S cm^−1^ for the bare-Li electrode and 1.73 × 10^−3^ S cm^−1^ for the BL-Li system. At 298.15 K, Li⁺ diffusion coefficients of 2.5 × 10^−10^ m^2^ s^−1^ (bare-Li) and 7.1 × 10^−10^ m^2^ s^−1^ (BL-Li) were obtained via the Nernst–Einstein relation, D = *μ*RT/(zF). In this expression, μ represents ionic mobility, R the gas constant, T the absolute temperature, z the ionic charge number, and F Faraday’s constant.

## Results and Discussion

### Bilayer Layout and Material Properties

**Fig. 1:**
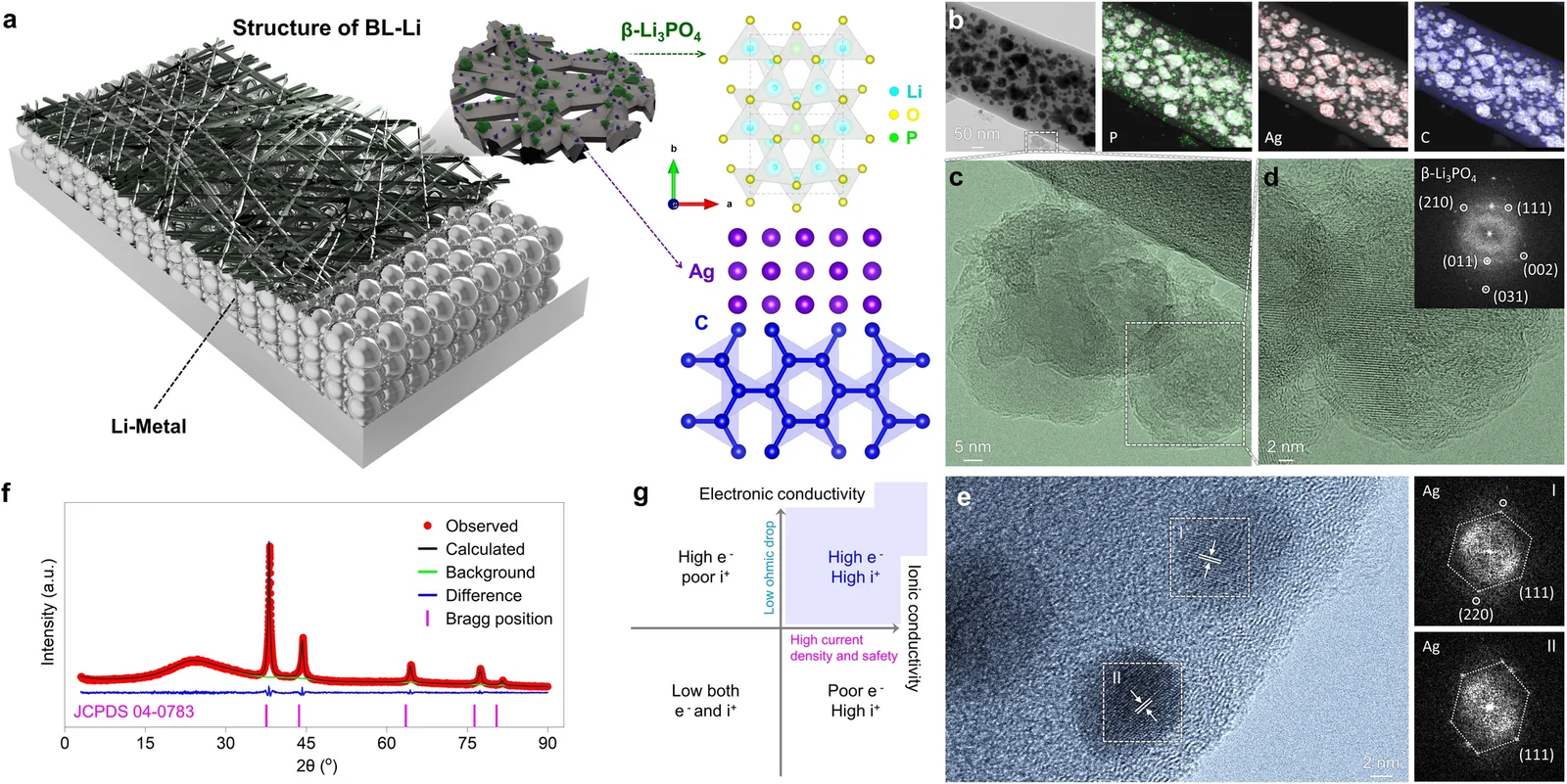
Schematic and material characterization of bilayer. **a** Conceptual illustration of BL-Li for Li anode. TEM images of **b** TEM image and EDX elemental maps of the BL layer, showing the distributions of P, Ag, and C. **c** and **d** High-resolution TEM images of the fiber component in BL; the inset in **d** is the FFT pattern. **e** High-resolution TEM image of Ag nanoparticles with FFT patterns shown in the insets. **f** XRD patterns of bilayer. **g** Quadrant chart of high e^−^ versus high i^+^ as a function of electronic and ionic conductivity

The bilayer comprises an Ag/C fibrous layer on the lithium-facing side and *β*-Li_3_PO_4_ particles on the electrolyte-facing side (Fig. 1a). The as-prepared electrically conductive layer (C-layer) was obtained by electrospinning an AgNO_3_/polyacrylonitrile (PAN) matrix, followed by the electrodeposition of a Li_3_PO_4_/polystyrene matrix to yield the as-prepared lithiophilic layer (L-layer). Polystyrene cross-links began to decompose at approximately 300 °C, whereas thermal degradation into styrene monomers and the volatilization of the oligomeric fragments occurred between 350 and 400 °C. Above 450 °C, most of the polystyrene was removed [33]. Consequently, a freestanding BL sheet was recovered in which only Li_3_PO_4_ adhered stably to the C-layer without a binder. To verify complete polystyrene removal, an L-layer-only sheet was subjected to identical thermal treatment. No trace of polystyrene was observed on the alumina plate that supported the sample, confirming the total loss of polymer. This finding further demonstrates that Li_3_PO_4_ can be effectively fixed on the C-layer surface without requiring an additional bonding process (Fig. S1). In Fig. 1b, the transmission electron microscope (TEM) elemental mapping of BL reveals a strong P signal at the lower exterior of the fiber, indicating that Li_3_PO_4_-like nanoparticles are firmly attached to the fiber surface. Notably, this sample withstood 30 min of ultrasonication before TEM grid loading and subsequent drop-casting. Following ultrasonication, the Li_3_PO_4_ particles remained firmly bound, demonstrating excellent adhesion durability. The Ag map further exhibited a dense distribution of Ag nanoparticles embedded within the carbon fiber, enriching electronic conduction pathways. Figure 1c shows a high-magnification view of the P-rich region, as previously shown in Fig. 1b, revealing Li_3_PO_4_ particles tens of nanometers in size. High-resolution imaging and inverse fast Fourier transform (IFFT) analyses, as shown in Fig. 1d, identify these particles as *β*-Li_3_PO_4_ with exposed (210), (011), (111), (002), and (031) facets [34, 35]. The commercially supplied Li_3_PO_4_ was initially amorphous but crystallized into β-Li_3_PO_4_ above approximately 600 °C during thermal treatment [35]. β-Li_3_PO_4_ offered high thermal and chemical stabilities and exhibited room-temperature ionic conductivities of the order of 10^−6^–10^−5^ S cm^−1^ in its crystalline form, providing widened diffusion channels for Li^+^ [36]. Enlarged Ag maps are shown in Fig. 1e, where spot 1 exhibits Ag(111) and Ag(220) reflections, whereas spot 2 exhibits only Ag(111) reflection. The L-layer side of the BL shows relatively darker surface features, while the C-layer presents a smooth and reflective appearance (Fig. S2). Scanning electron microscope (SEM) and energy-dispersive X-ray spectroscopy (EDX) mapping were performed to analyze the bilayer morphology and elemental distribution. In the SEM/EDX images of the L-layer direction (Fig. S3), particles ranging from several micrometers up to hundreds of micrometers are uniformly distributed across the fiber network. The SEM image of the C-layer surface (Fig. S4) reveals much smaller particles of 100–200 nm in size, which are identified as Ag nanoparticles by elemental mapping. The cross-sectional SEM image of the bilayer (Fig. S5) shows a total thickness of approximately 3.5 μm. And, the bilayer structure was confirmed by cross-sectional SEM–EDS mapping (Fig. S6). To elucidate how Li₃PO₄ is anchored to the Ag/C fibers, additional HAADF-STEM and EDS analyses were performed (Fig. S7). The results reveal that silver particles are located inside the carbon fibers, whereas P elements are distributed around and cover the surfaces of the carbon fibers. In addition, P-containing agglomerates with sizes on the order of several hundred nanometers were observed to be attached to the surfaces of the carbon fibers. High-magnification HAADF-STEM observations of these agglomerates further indicate that the P elements are encapsulated by carbon. Figure S8 presents the TEM image corresponding to Fig. S7, showing the agglomerate region at higher magnification. TEM analysis confirms that the Li_3_PO_4_ domains are surrounded by graphitic layers (Fig. S9). Taken together, these results demonstrate that, as confirmed by SEM–EDS analysis, Li_3_PO_4_ is abundantly present on the upper surface of the BL, and that Li_3_PO_4_ is attached around the Ag/C fibers in the form of agglomerates with sizes of several hundred nanometers. This structural feature suggests that, during the thermal decomposition of polystyrene, most of the polymer matrix is removed, while a small fraction of carbon remains and forms graphitic layers, which in turn firmly anchor Li_3_PO_4_ to the Ag/C fibers.

Figure 1f shows the XRD pattern measured from the L-layer side of the BL sheet, with the intense Ag peaks confirming the presence of silver (front and back measurements are provided in Fig. S10). The synthesis process for BL is represented by Eqs. 4 and 5

L-layer:

4 $$\begin{aligned} &{\mathrm{Li}}_{{3}} {\mathrm{PO}}_{{4}} \left( {\mathrm{s}} \right) + \left( {{\mathrm{C}}_{{8}} {\mathrm{H}}_{{8}} } \right)_{{\mathrm{n}}} \, \to \, \beta - {\mathrm{Li}}_{{3}} {\mathrm{PO}}_{{4}} \left( {\mathrm{s}} \right) + {\mathrm{nC}}_{{8}} {\mathrm{H}}_{{8}} \left( {\mathrm{g}} \right) \\ & \quad + {\text{Volatile products}} + {\text{Trace carbon residues}} \\ \end{aligned}$$

C-layer:

$${\mathrm{2AgNO}}_{{3}} \left( {\mathrm{s}} \right) \to {\text{2Ag }}\left( {\mathrm{s}} \right) + {\mathrm{2NO}}_{{2}} \left( {\mathrm{g}} \right) + {\mathrm{O}}_{{2}} \left( {\mathrm{g}} \right)$$

5 $$\left( {{\mathrm{C}}_{{3}} {\mathrm{H}}_{{3}} {\mathrm{N}}} \right)_{{\mathrm{n}}} \to {\text{ C}}_{{\mathrm{n}}} \left( {\text{Carbon fiber}} \right) + {\text{Volatile products}}$$

The high conductivity of ASEI was attributed to the uniform distribution of carbon fiber sheets with Ag nanoparticles. Maximizing electronic conductivity in the ASEI reduces the internal resistance (R) of the electrode/current collector, effectively decreasing the ohmic drop (Δ*V* = *I* × *R*). *β*-Li_3_PO_4_ can simultaneously lower bulk resistance (*R*_bulk_) and interfacial resistance (*R*_int_), substantially reducing the overall ionic conduction resistance (*R*_ion_) [25, 37] (Fig. 1g).

### Impact on Li Electrode Morphology and Surface

**Fig. 2:**
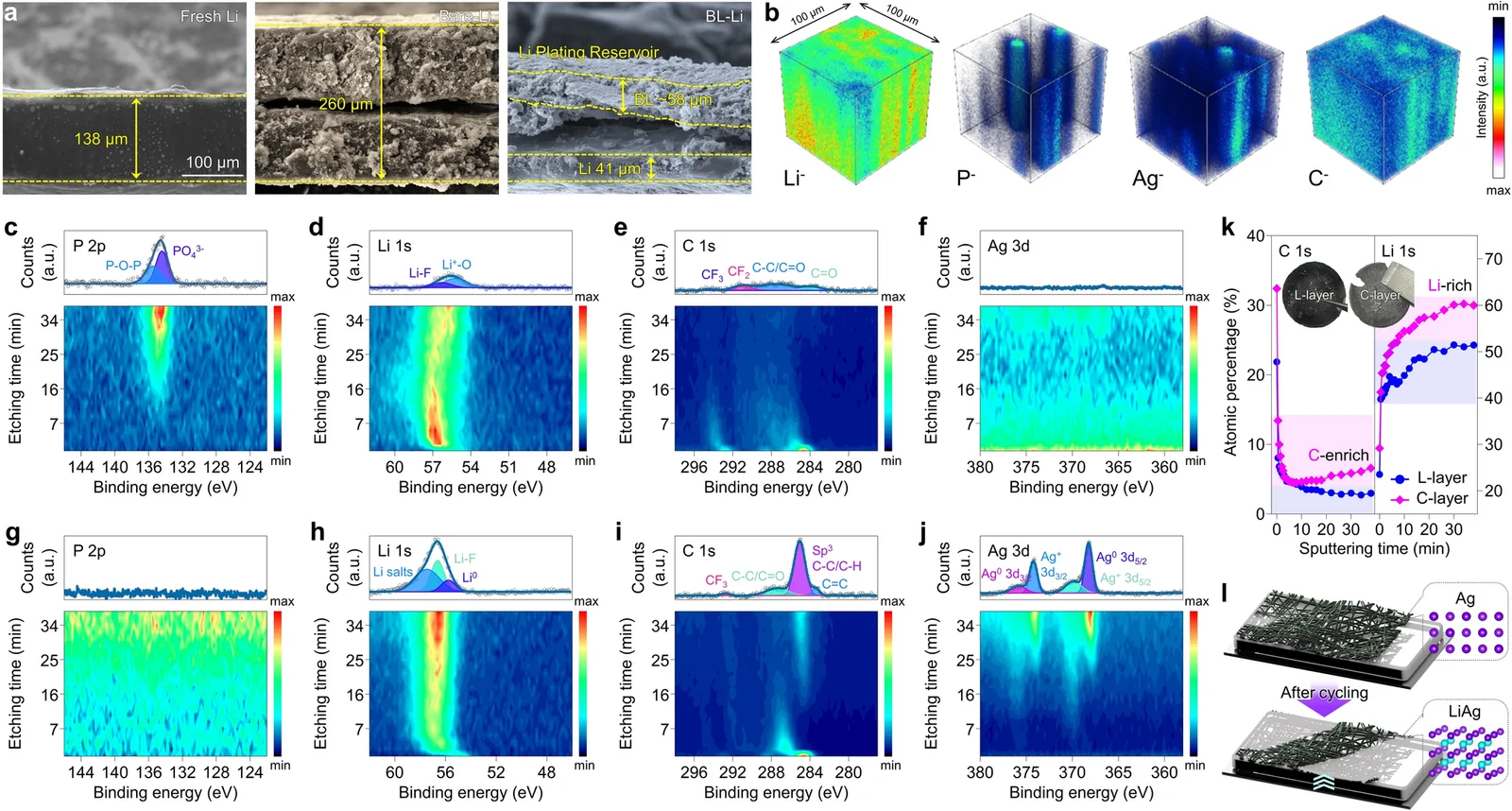
Experiments are performed for 100 cycles at 1 mA cm^−2^ and 1 mAh cm^−2^. **a** Cross-sectional SEM images of fresh-Li, Bare-Li, and BL-Li. **b** TOF–SIMS 3D rendering views of fragments distribution Li^−^, C^−^, Ag^−^, and P^−^. Depth-profiled XPS measurements of P 2*p*, Li 1*s*, C 1*s*, and Ag 3*d*: Front side (upper surface) of the bilayer at **c to f** and back side (bottom side) of the bilayer at **g to j**. Each plot comprises of two individual figures, i.e., the point after etching (up) and depth profiling spectrum (down). **k** XPS elemental analysis of BL/Li at C 1*s* and Li 1*s*. Inset: images of BL after cycling. **l** Illustration of formation biphasic-dependent Li deposition

To study the effects of ASEI during cycling, Li–Li symmetric cells are tested using chronoamperometry for 100 cycles at 1 mA cm^−2^ with a fixed capacity of 1 mAh cm^−2^, and post-mortem analyses are conducted, as shown in Fig. 2. Li metal symmetric cells are subjected to 100 plating/stripping cycles (Fig. S11). After cycling, the cells were disassembled to compare the thickness of the Li electrodes. The electrode thickness of the bare-Li symmetric cell increased to 260 μm compared to 138 μm for fresh-Li, whereas the electrode thickness of the BL-Li symmetric cell decreased significantly to approximately 100 μm. Fresh-Li foil measured 138 µm in thickness, whereas the BL-Li cell post-cycling thickness decreased slightly owing to compression during cell assembly. In the plane-view SEM images, the bare-Li electrode (Fig. S13a, b) shows an uneven surface, whereas the BL-Li electrode (Fig. S13c, d) exhibits a flat morphology. Post-cycle electrochemical impedance spectroscopy (EIS) reveals that the BL-Li electrode maintains significantly lower impedance without any evident increase, corroborating its role in preserving plated Li and supporting high-rate charge/discharge (Fig. S13). And Fig. S14 is Li electrode after 100 cycles at 1 mA cm^−2^ with a fixed capacity of 1 mAh cm^−2^.

The surface and cross-sectional morphologies of the Li electrodes after the first stripping and plating in Li–Li symmetric cells were analyzed using ex situ SEM, as shown in Fig. S15. For the bare-Li electrode, only one side was fixed as the positive electrode for observation, whereas for the BL-Li electrode, both the positive and negative electrodes were examined. During the first plating step, when a positive current is applied, electrons flow from the negative electrode to the positive electrode through the external circuit, resulting in Li plating at the positive electrode and Li stripping at the negative electrode. In the case of the bare-Li electrode, lithium dendritic growth was observed on the surface during plating (Fig. S15a, b), and the electrode thickness increased slightly from approximately 141 μm for fresh-Li (Fig. S16) to 143 μm after plating (Fig. S15a, c). In the BL-Li symmetric cell, Li stripping at the negative electrode led to a morphology, suggesting that the BL interior became progressively filled with lithium (Fig. S15d, e), accompanied by a decrease in the thickness of the lithium metal (Fig. S15f). Meanwhile, Li plating occurred at the positive electrode. Plane-view SEM images reveal that the fibrous structure became more clearly exposed compared to the stripping state (Fig. S15g, h), and the electrode thickness increased accordingly (Fig. S15i). Subsequently, during the stripping step with reversed polarity, Li plating occurred at the negative electrode and Li stripping at the positive electrode. For the bare-Li electrode, pronounced tortuous dendritic morphologies were clearly observed on the surface after stripping (Fig. S15j, k), along with a further increase in electrode thickness (Fig. S15l). For the BL-Li electrode, Li plating at the negative electrode resulted in a surface morphology characterized by partially exposed fine granular features (Fig. S15m, n), and the electrode thickness increased slightly from 99.5 to 102.1 μm (Fig. S15o). In contrast, Li stripping at the positive electrode produced a morphology in which the BL interior appeared densified, similar to that observed at the negative electrode during the plating stage (Fig. S15p, q), and the electrode thickness decreased from 104.7 to 99.4 μm (Fig. S15r). Overall, these results indicate that, in the BL-Li electrode, the BL interior becomes denser during Li stripping, whereas the fibrous morphology is more clearly revealed during Li plating. This behavior is consistent with the preferential lithium deposition toward the C-layer direction observed in Fig. 2c–j. In addition, repeated plating and stripping lead to a gradual increase in the thickness of the BL, while the thickness of the lithium metal itself gradually decreases. Therefore, the significantly reduced cross-sectional thickness of the BL-Li electrode shown in Fig. 2a, compared to that of fresh-Li (138 μm), can be attributed to the progressive ionization and storage of lithium within the BL during repeated stripping and plating processes. This behavior suggests that Ag nanoparticles anchored on the carbon fibers repeatedly participate in Li–Ag alloying and Li deposition reactions, enabling the BL to function as an effective lithium-plating reservoir. Meanwhile, the apparent gap observed between the BL and the lithium metal originates from the intrinsic material characteristics of the carbon fiber-based structure that constitutes the BL. Owing to its fibrous internal network, the BL exhibits excellent wettability toward liquids such as electrolytes, and in a wetted state, it readily adheres to various substrates (Fig. S17a). In contrast, under dry conditions, the BL maintains a very thin, lightweight, and freestanding morphology (Fig. S17b). Therefore, in the high-vacuum environment of the SEM chamber, where the BL becomes completely dry, it tends to retain its original shape, making it appear separated from the lithium surface.

Time-of-Flight secondary ion mass spectrometry (TOF–SIMS) surface mapping of the Li metal anode (Fig. 2b) shows higher Li intensity in regions of dense Ag signal, whereas Li_3_PO_4_ does not exhibit any evident spatial correlation with Li. In addition, it was detected only in minor amounts. This limitation arises because the sputtering depth of the instrument is generally restricted to several tens to several hundreds of nanometers, which is insufficient to clearly resolve the two separated layers of the BL. Figure S18 shows the TOF–SIMS depth profile. Throughout sputtering, the Li^−^, C^−^, Ag^−^, and P^−^ ion intensities formed a uniform plateau, and their relative magnitudes indicated that the elemental distribution was in the order Li > C > Ag > P.

In situ X-ray photoelectron spectroscopy (XPS) was performed to identify the chemical states of the Li metal anode. Figure 2c–f shows the L-layer spectra, and Fig. 2g–j shows the C-layer spectra before and after Ar^+^ sputtering, where the overlaid profiles correspond to post-sputtering conditions. In Fig. 2c, the P 2*p* signal confirms the presence of PO_4_^3−^ and P–O–P bonds, corresponding to Li_3_PO_4_ even after partial etching [38]. The Li 1*s* spectra in Fig. 2d show an initial peak near 57 eV, corresponding to electrolyte decomposition products that diminish with sputtering. However, the re-emergence of Li^+^–O bonding at P 2*p* onset in the final spectrum further indicates intact Li_3_PO_4_ [39]. Following the decomposition of Li_3_PO_4_ to Li_3_P + 4 Li_2_O, a Li–P peak should be observed in the range of 133.5–134.5 eV in the P 2*p* region, which was absent in this study. The Li–F feature in Li 1*s* arises from Ar^+^-induced lithium bis(trifluoromethanesulfonyl)imide (LiTFSI) decomposition [40]. In C 1*s*, early spectra detected C=O [41] and CF_3_ [42] bonds, whereas final spectra revealed CF_2_ (291.5 eV) and –CF_3_ species from LiTFSI breakdown [42]. No notable bonding changes were detected for Ag 3*d*. In the C-layer, P 2*p* was absent. Initially, Li 1*s* exhibited peaks corresponding to Li^0^ and Li–F. Following sputtering, the peaks corresponding to electrolyte decomposition (Li salts), Li–F, and Li^0^ were observed, which were consistent with the expected results from direct Li contact and LiTFSI breakdown [40, 43]. C 1*s* early spectra featured enhanced *sp*^3^ bonding, transitioning to CF_3_ [42], C–C/C=O [41], *sp*^3^ [44], and finally to C=C [45]. The peak intensity corresponding to Ag 3*d* increases concurrently with Li 1*s*, signifying Ag-Li chemical interactions [43, 46, 47]. Figure 2k images (left: L-layer; right: C-layer) and depth profiles show that C 1*s* exposure increases significantly in the C-layer with sputtering time compared to L-layer, and Li 1s atomic percentage in the C-layer exceeds that in the L-layer progressively. These findings confirmed preferential Li deposition near the C-layer and designated the L-layer as an ion transport conduit (Fig. 2l).

### Monitoring Li Plating Patterns

**Fig. 3:**
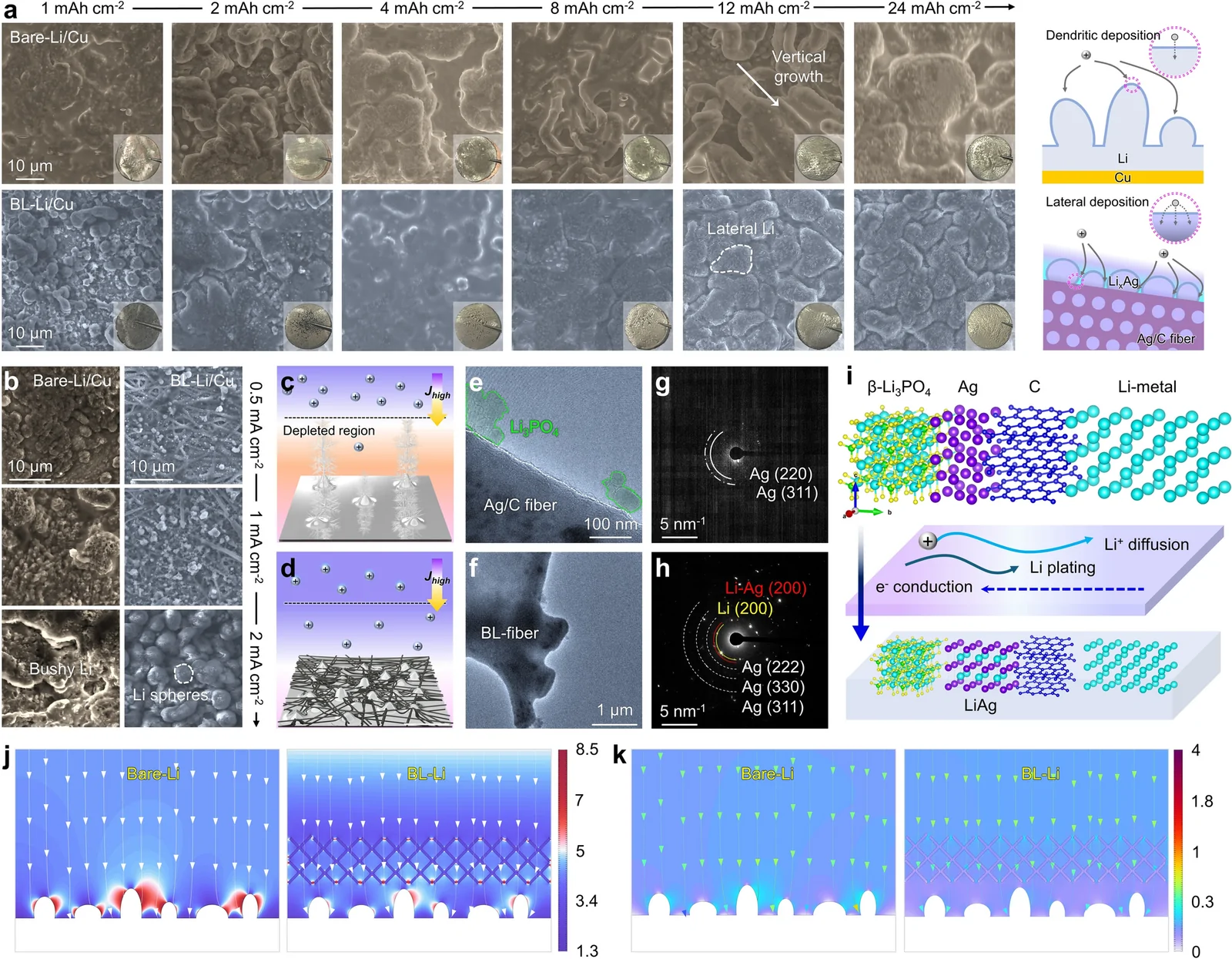
Screening of mechanisms and structural effects. **a** Digital photograph (inset) and corresponding SEM images for bare-Cu and BL-Cu in asymmetric Cu-Li cells under operating conditions of 1 mA cm^−2^ for 1, 2, 4, 8, 12, and 24h (from left to right) with schematics of the cross-sectional morphologies. **b** SEM images for bare-Cu and BL-Cu in asymmetric Cu–Li cells under operating conditions of 0.5, 1, and 2 mAh cm^−2^ for 0.5 h. Cross-sectional illustrations of Li deposits on** c** bare-Cu and **d** BL-Cu under the operating conditions of **b** Cryo-TEM images for grown Li at 1 mA cm^−2^ for 1h on BL-Cu **e and g** in asymmetric Cu–Li cells. Corresponding selected area electron diffraction patterns of bilayer modified Cu in asymmetric Cu–Li cell are shown in **f and h**. **i** Schematic of Li deposition behavior of BL system. **j and k** COMSOL Multiphysics simulations of bare-Li and BL–Li

To monitor the Li plating process, an asymmetric Li–Cu cell configuration was used. The bare-Cu electrode cell exhibited vertical Li growth, characteristic of dendrite formation, with increasing areal charge capacity. However, the Cu electrode cell incorporating the BL structure initially formed spherical Li deposits that subsequently flattened, transitioning to a lateral plating morphology once the areal charge capacity reached 12 mAh cm^−2^. Vertical growth on the bare-Cu electrode was attributed to the low mutual solubility between Cu and Li. The high nucleation overpotential confined Li nucleation to a few active sites, which rapidly developed into dendritic structures [48]. Micro-roughness and heterogeneities on the Cu surface further concentrated the local current density, causing concentration polarization of Li^+^ and intensifying electric fields at dendrite tips, leading to self-accelerating protrusion growth [49]. In contrast, Ag nanoparticles decreased the nucleation energy of Li via the wide solid-solution region, as shown in the Ag-Li phase diagram [50]. The decreased nucleation overpotential facilitated adsorption and uniform distribution of Li^+^ on Ag surfaces, creating multiple homogeneously distributed nuclei that develop into smooth masses through subsequent Ag-Li solid-solution alloying [50]. Digital photographs confirmed that although the bare-Cu electrode exhibited irregular Li patches, the BL-Cu electrode filled its three-dimensional fiber network with Li, which served as a matrix. The carbon-Ag fiber scaffold enveloped spherical and tiny dendritic features, maintaining continuous electronic percolation paths irrespective of deposition sites. In addition, lithium was plated in asymmetric Li–Cu cells to capacities of 1, 2, 4, 8, and 24 mAh cm^−2^, after which the BL was removed and the Cu surface was washed with DME prior to SEM observation (Fig. S19). As observed in the SEM images, no distinct morphological features attributable to lithium deposition were detected on the Cu surface, a trend that becomes more evident when compared with the results observed on the BL side of the BL–Li/Cu electrode shown in Fig. 3a. To further verify the possibility that lithium ions may bypass the BL and directly nucleate on the underlying substrate, optical images obtained after lithium plating to a capacity of 24 mAh cm^−2^ in a Cu–Li asymmetric cell configuration are presented in Fig. S20. The images display the L-layer side of the BL (Fig. S20a) and the C-layer side of the BL (Fig. S20b), respectively. Visual inspection after plating revealed a substantial amount of lithium on the BL, whereas the underlying Cu current collector did not exhibit the characteristic silvery metallic luster typically associated with lithium deposition. Furthermore, in the same Cu–Li asymmetric configuration, lithium was plated to capacities of 4 and 24 mAh cm^−2^, and cross-sectional SEM images of the resulting BL–Li/Cu electrodes were examined (Fig. S21). The results indicate that lithium preferentially and densely deposits toward the C-layer side of the bilayer structure. Furthermore, the reversibility of plated Li is assessed by stripping the same areal capacity (24 mAh cm^−2^), as shown in Fig. S22. The bare Cu electrode exhibited dark residues on its surface, likely attributed to the presence of electrolyte decomposition products or electrochemically inactive “dead Li”. In contrast, although the BL-Cu electrode fractured during cell disassembly, no visible Li residues remained, suggesting that Li plated on the BL-Cu electrode underwent more complete electronic reionization and reversible stripping compared to the bare-Cu electrode.

To investigate the effect of current density on Li plating morphology, the plating time was fixed at 30 min with current densities of 0.5, 1.0, and 2.0 mA cm^−2^. In the bare-Cu cell, low-rate plating at 0.5 mA cm^−2^ produced Li deposits several micrometers in size, but as current density increased, deposition became more uneven and bushy dendritic structures dominated. In contrast, the BL-Cu cell exhibited uniformly distributed submicron Li deposits on Ag nanoparticles at 0.5 mA cm^−2^, resulting in an overall thickening of the fiber scaffold. At 1.0 mA cm^−2^, the particle size grew further, and at 2.0 mA cm^−2^ the fiber morphology disappeared entirely, which was replaced by spherical Li particles. This behavior stems from the fact that higher current densities increase overpotential and accelerate nucleation rates, rapidly consuming Li^+^ and intensifying concentration polarization in the electrolyte. In the bare-Cu cell, the process becomes diffusion-limited, depleting Li^+^ locally and leading to anisotropic, bushy dendrite growth (Fig. 3c). Conversely, the uniformly dispersed Ag nanoparticles in the BL scaffold homogenize the local current density (Fig. 3d). A uniform current distribution allows Li^+^ to reach all surfaces evenly, promoting isotropic growth and yielding smooth, spherical deposits even under high-rate conditions [51, 52].

Cryo-ultramicrotomy was performed to verify Li-Ag alloy formation within the BL scaffold. Figure 3e, f shows the cryosectioned exterior of a BL fiber, where Li_3_PO_4_ particles anchored to the fiber surface, as previously observed in Fig. 1c. Selected area electron diffraction (SAED) patterns acquired from this region exhibited evident Ag(220) and Ag(311) reflections (Fig. 3g) [53] Low-magnification imaging of the overall BL architecture followed by SAED analysis revealed diffraction from Li(200) [43], Li-Ag(200) [43], as well as Ag(311) [53], Ag(330) [54], and Ag(222) [55] planes (Fig. 3h). The following reactions are proposed for the Ag-Li system within the BL, as given by Eq. 6 [43]:

$${\text{Ag }}\left( {\mathrm{s}} \right) + {\text{xLi }}\left( {\mathrm{s}} \right) \rightleftharpoons {\text{LixAg }}\left( {\mathrm{s}} \right)$$

$${\text{Ag }}\left( {\mathrm{s}} \right) + {\mathrm{xLi}}^{ + } + {\mathrm{xe}}^{ - } \to {\text{ Li}}_{{\mathrm{x}}} {\text{Ag }}\left( {\mathrm{s}} \right), \, (0 < {\mathrm{x}} \le 0.{4})$$

$${\mathrm{Li}}_{{\mathrm{x}}} {\text{Ag }}\left( {\mathrm{s}} \right) + ({1} - {\mathrm{x}}){\mathrm{Li}}^{ + } + ({1} - {\mathrm{x}}){\text{ e}}^{ - } \to {\text{ LiAg }}\left( {\mathrm{s}} \right)$$

$${\text{LiAg }}\left( {\mathrm{s}} \right) + {\mathrm{Li}}^{ + } + {\mathrm{e}}^{ - } \to {\text{ Li }}\left( {\mathrm{s}} \right) + {\text{Ag }}\left( {\mathrm{s}} \right)$$

6 $${\text{Li }}\left( {\mathrm{s}} \right) + {\mathrm{Li}}^{ + } + {\mathrm{e}}^{ - } \to {\text{ LiAg }}\left( {\mathrm{s}} \right) + {\mathrm{Li}}_{{\mathrm{x}}} {\text{Ag }}\left( {\mathrm{s}} \right) \, \to {\text{ Ag }}\left( {\mathrm{s}} \right)$$

Furthermore, finite-element simulations using COMSOL Multiphysics were conducted to map Li⁺ ion flux (Fig. 3j) and electrolyte current density distribution (Fig. 3k) at the electrode interface. On the bare-Li electrode, pronounced flux funneling toward microscopic asperities creates localized current density hotspots that accelerate dendritic tip growth. In contrast, the bilayer disperses Li⁺ flux uniformly across the interface and smooths out the current distribution. This mitigation of ionic and electronic hotspots lowers local overpotentials and suppresses preferential dendritic nucleation, yielding a more uniform Li deposit morphology during self-repair cycles.

### Study of Electrochemical Behavior

To assess the critical current density of the engineered solid–electrolyte interphase, Li–Li symmetric cells were tested at a fixed areal charge capacity of 1 mAh cm^−2^ at varying current densities from 0.5 to 20 mA cm^−2^. The bare-Li cell maintained a symmetric voltage profile up to 4 mA cm^−2^, but exhibited enhanced asymmetry at 6 mA cm^−2^ and immediate short-circuiting at 10 mA cm^−2^. In contrast, the BL-Li cell operated stably even at 20 mA cm^−2^, exhibiting an exceptionally low overpotential of approximately 99 mV at the mid-plateau. In addition, in a lithium symmetric cell configuration, BL–Li electrodes were cycled under conditions of 1 mA cm^−2^ and 1 mAh cm^−2^. After both the first cycle and the 10th cycle, the BL was removed following stripping and plating, and the exposed Li surface was examined by SEM (Fig. S23). In both cases, no prominent lithium deposition or reaction features were observed on the Li side of the BL–Li electrode. These results demonstrate that the BL–Li system effectively suppresses lithium-ion bypassing through the BL and subsequent direct nucleation on the lithium metal surface. Under extreme conditions (20 mA cm^−2^, 10 mAh cm^−2^), 300 h of plating/stripping cycling rendered the bare-Li cell inoperable attributed to voltage spikes above 5 V, whereas the BL-Li cell sustained overpotentials of 120 mV initially, 111 mV at 100 h, 106 mV at 200 h, and 118 mV at 300 h, demonstrating outstanding durability. In general, the occurrence of an internal short circuit in a cell is accompanied by localized darkened traces on the separator and excessive lithium dendrite growth near the corresponding electrode region. In the BL-based cell, inspection of the separator after approximately 20 h of operation revealed no such darkened marks, and no locally abnormal lithium deposition was observed on the electrode surface (Fig. S24a, b). In contrast, after prolonged cycling (≈320h), darkened traces were detected on the separator, accompanied by excessive lithium accumulation at spatially corresponding regions of the electrode (Fig. S24c, d). Furthermore, after approximately 350 h, a rapid increase in overpotential followed by cell failure was observed, which is attributed to an internal short circuit. These ex situ observations indicate that although the BL/Li system can delay short-circuit formation under high current density conditions, prolonged cycling may eventually lead to localized lithium accumulation and short-circuit failure. Figure S25 presents the initial voltage–time profile obtained at 20 mA cm^−2^ with a cumulative areal capacity of 10 mAh cm^−2^. The pronounced tailing behavior observed in the negative voltage region suggests that, under such high current density conditions, lithium stripping is more strongly limited by mass transport compared to lithium plating. To further examine whether a short circuit had already occurred under these conditions, GITT measurements were conducted using a lithium symmetric cell configuration (Fig. S26). Although relatively high polarization voltages were observed, the simultaneously monitored cell resistance remained within the range of approximately 80–120 Ω, indicating that a hard short circuit was not present during the measurement. Moreover, Li|Li symmetric cells are tested at a low current density of 1 mA cm^−2^ and an areal capacity of 1 mAh cm^−2^ (Fig. S27). The bare‐Li cell exhibited overpotentials of 52, 81, and 73 mV at 100, 300, and 500 h, respectively. In contrast, the BL‐Li cell maintained low overpotentials of 9.5, 9.52, 12, and 12 mV at 100, 300, 500, and 800 h, respectively. These results demonstrate that, even under low current‐density conditions, the BL‐Li anode can sustain minimal polarization over extended cycling. Galvanostatic intermittent titration measurements with current pulses yielded initial plating Δ*V*₀ values of 0.26, 0.11, and 0.09 V (bare-Li) vs. 0.06, 0.05, and 0.05 V (BL–Li) and stripping values of 0.08, 0.06, 0.14 V (bare-Li) vs. 0.04, 0.03, and 0.05 V (BL–Li), respectively. Calculated area-specific resistances (Ω cm^2^) via *R*ₛ = Δ*V*₀/*I* are 168.9, 71.5, 56.5, 53.9, 40.9, 90.9 for bare-Li and 41.6, 35.1, 32.5, 22.7, 18.2, 33.8 for BL-Li. The remarkably lower IR drop in the BL–Li cell indicated significantly reduced pure ohmic resistance, indicating enhanced ionic conductivity and minimized SEI/interfacial resistances. These findings suggest that the architecture of BL ASEI maintains low overpotential and low resistance even at high current densities, minimizing voltage loss, heat generation, and Coulombic inefficiency, which are key factors for enabling long-life Li metal battery operation at high power.

**Fig. 4:**
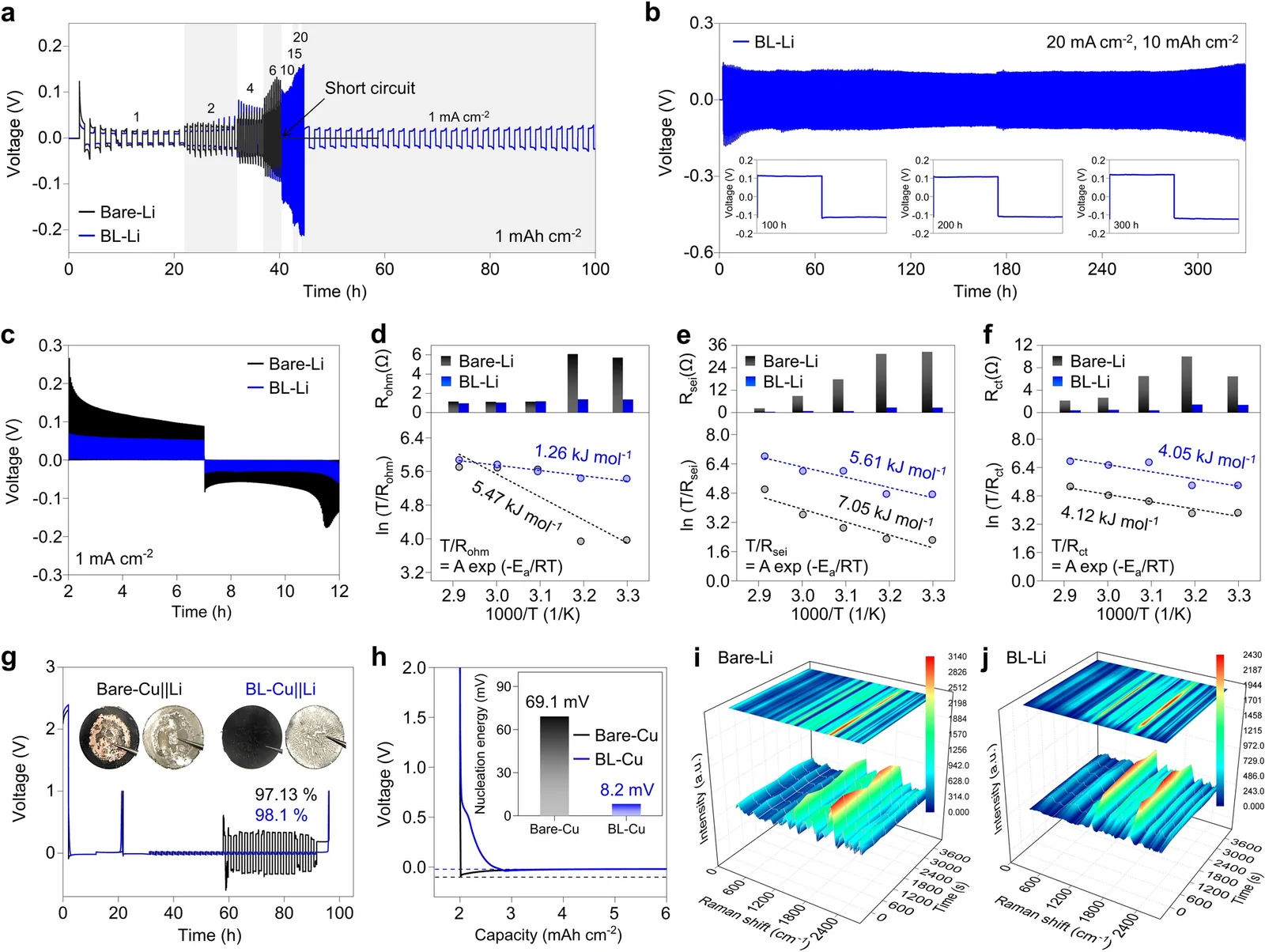
Study of electrochemical behavior in Li–Li symmetric cell configuration. Galvanostatic charge–discharge voltage profiles of Li–Li symmetric cells with operating conditions at varying current density with a fixed capacity of 1 mAh cm^−2^ **a** and at 20 mA cm^−2^, 10  mAh cm^−2^ over 300 h **b**. **c** Galvanostatic intermittent titration (GITT) profiles at 1 mA cm^−2^ of bare-Li and BL-Li cells. Arrhenius curves and calculated activation energy dependence of **d** *R*_ohm_, **e** *R*_sei_, and **f** *R*_ct_ in the symmetric bare-Li and BL-Li cells. **g** Time–voltage profiles of Lil||Cu cells with pristine Cu and bilayer modified Cu measured via Aurbach method. **h** Voltage vs. time graph of Li plating at a current density of 0.5 mA cm^−2^ with inset exhibiting Li nucleation energy of bare-Li and BL–Li cells. In situ Raman spectra of **i** bare-Li and **j** BL–Li in Li–Li symmetric cells at a current density of 2 mA cm^−2^ and a capacity of 1 mAh cm^−2^

To elucidate the relationship between Li-ion mobility and electrode kinetics, activation energies (*E*ₐ) are extracted via Arrhenius analysis of temperature-dependent EIS data (Fig. S28). Compared to the bare-Li symmetric cell, the BL-Li cell exhibited reduced *E*ₐ for Ohmic resistance (*R*ₒₕₘ), SEI resistance (*R*ₛₑᵢ), and charge-transfer resistance (*R*_ct_). The significant decrease in *R*ₒₕₘ *E*ₐ indicates that BL effectively lowers the ion diffusion barrier within the SEI (Fig. 4d). The substantial reduction in SEI resistance *E*ₐ, formerly the rate-determining step in the bare-Li cell, confirms that the BL decreases the interfacial reaction energy barrier (Fig. 4e). Similarly, the reduced *R*_ct_ *E*ₐ in the BL-Li cell facilitates charge transfer at the electrode/electrolyte interface (Fig. 4f). Tafel plots yield exchange current densities (*j*₀) of 0.59 mA cm^−2^ for bare-Li and 0.66 mA cm^−2^ for BL–Li (Fig. S29). Ionic conductivities increase from 7.68 × 10^−5^ S cm^−1^ (bare-Li) to 1.73 × 10^−3^ S cm^−1^ (BL-Li) (Fig. S30), and transference numbers increase from 0.47 to 0.66, indicating enhanced Li^+^ transport (Fig. S31). Post-polarization EIS exhibited minimal impedance change for BL-Li, evidencing robustness against polarization stress. Comparing C-layer-Li and BL-Li cells (Fig. S32), the lower ionic conductivity (1.07 × 10^−3^ S cm^−1^) and transference number (0.12) of C-layer-Li cell highlight the role of the Li_3_PO_4_ upper layer in reducing ion transport resistance. In addition, to more clearly elucidate the role of Li_3_PO_4_ in the BL system, comparative experiments were performed using lithium symmetric cells in Fig. S33. To distinguish the effect of Li_3_PO_4_, lithium symmetric cell tests were conducted at 4 mA cm^−2^ and 1 mAh cm^−2^ using four different configurations: an ASEI consisting solely of carbon fibers (C-only), Li₃PO₄ combined with carbon fibers (Li_3_PO_4_ + C-only), a BL with the stacking orientation reversed (BL reverse), and the original BL configuration. In lithium symmetric cells, the potential difference between the plateaus of the positive and negative curves is commonly referred to as the overpotential or Ohmic component, while the regions outside the plateau are generally considered to be dominated by mass transfer processes [56]. The C-only, Li_3_PO_4_ + C-only, and BL reverse configurations all exhibit pronounced tailing behavior in the mass-transfer-dominated region, whereas this behavior is significantly suppressed in the BL system. It has been reported that tailing behavior observed in lithium symmetric cell tests can be associated with lithium-ion concentration gradients between the electrodes [57]. In other words, insufficient mass transport can lead to increased overpotential, resulting in tailing behavior. When the electrically conductive layer is predominantly located on the upper side, lithium tends to deposit preferentially on the upper surface according to its potential energy [58]. This configuration can increase tortuosity, thereby hindering lithium-ion transport toward the electrode and ultimately impeding mass transfer. In addition, in the absence of strategies such as intermetallic alloying with lithium to reduce overpotential, achieving uniform lithium deposition may also be challenging. Consistent with this interpretation, the C-only based configuration without Ag exhibits particularly high overpotential during the lithium stripping process, whereas the introduction of a bilayer structure alone leads to a noticeable reduction in this effect. Nevertheless, our results further indicate that the bilayer structure must be designed to promote lithium deposition toward the upper side, thereby minimizing lithium-ion concentration imbalance and enabling more efficient mass transport, as suggested in the present study. Importantly, although the two symmetric cells contain identical components, the interfacial stacking orientation within each half-cell remains fixed throughout cycling. While lithium plating and stripping alternate between the two electrodes in a symmetric cell configuration, the internal bilayer architecture of each electrode does not change. Therefore, the Li⁺ flux distribution and the preferential nucleation sites determined by the stacking orientation are not averaged out during cycling. Instead, these structural differences continuously influence local current density distribution and ion transport pathways. After completion of the experimental conditions, the cells were disassembled, the ASEI layers were removed, and the Li surfaces were examined by SEM. Considering that these observations were made after 50 cycles under a relatively high current density of 4 mA cm^−2^ and an areal capacity of 1 mAh cm^−2^, the surfaces of all samples appeared generally clean. Nevertheless, a comparative assessment of the surface morphologies reveals that the degree of surface cleanliness follows a trend consistent with the electrochemical behavior observed in Fig. S34. In addition, to quantitatively evaluate the lithium-ion diffusion characteristics provided by Li_3_PO_4_ with respect to the stacking orientation of the BL, GITT analyses were further conducted using lithium symmetric cells (Fig. S35). In GITT measurements, a lithium-ion diffusivity is calculated for each current pulse; therefore, in this study, the diffusivity values obtained from all pulses were compiled, and the median value was used as a representative parameter for comparison. The results show that the forward BL configuration exhibits a lower IR drop than the BL (reverse) configuration, and the effective Li⁺ diffusivity derived from the voltage response is also higher (forward BL: ~ 1.9 × 10^−10^ cm^2^ s^−1^; BL (reverse): ~ 5.2 × 10^−11^ cm^2^ s^−1^). These results suggest that the forward BL structure provides more favorable conditions for Li⁺ transport. Such differences in diffusion characteristics are expected to progressively influence the internal cell environment and long-term stability upon prolonged cycling, and the present results suggest that the BL system proposed in this study has been designed and evaluated under a relatively optimized stacking orientation.

In asymmetric Cu–Li cells tested at 1 mA cm^−2^ and 1 mAh cm^−2^ (Fig. S36), the bare-Cu cell’s Coulombic efficiency decreases after 20 cycles, whereas the BL-Cu cell maintains 97% efficiency over 100 cycles with negligible change in voltage–capacity profiles from cycle 2 to 90.

The lifetime lost capacity of the asymmetric Cu–Li cells is calculated using the Aurbach method [56] (Fig. 4g). The bare-Cu cell’s voltage profile became unstable after approximately 60 h, likely attributed to accumulated dead Li and electrolyte side products degrading the electrode surface and causing a sudden rise in overpotential. Post-mortem examination revealed a thick layer of dark residue on the Cu current collector and severe morphological degradation of the Li metal counter electrode. In contrast, the BL-Cu cell maintained a stable voltage profile throughout testing and achieved a Coulombic efficiency of 98.10%, compared with 97.13% for the bare-Cu cell, demonstrating that the BL architecture significantly reduced lifetime lost capacity. No significant degradation of the Li surface was observed on the BL-Cu electrode post-test disassembly.

Nucleation barriers at the initial Li reservoir stage are measured (Fig. 4h). The bare-Cu cell exhibited a barrier of 69.1 mV, whereas the BL-Cu cell exhibited a significantly lower barrier of only 8.2 mV. This substantial reduction arises from uniform Li^+^ adsorption and multistep Ag-Li alloying on the Ag nanoparticle surface. Upon desolvation, Li^+^ forms a thin Li_x_Ag intermediate on Ag, then converts to LiAg, and finally nucleates as metallic Li [57, 58]. The intermetallic compound layers lower the subsequent Li diffusion barrier, preventing local supersaturation-induced dendrites and promoting lateral plating [57, 58]. The overall mechanism can be summarized as:

- (1) Nucleation Step
  $${\text{Ag }}\left( {\mathrm{s}} \right) + {\text{x Li}}^{ + } + {\text{x e}}^{ - } \to {\text{ Li}}_{{\mathrm{x}}} {\text{Ag }}\left( {\mathrm{s}} \right) \, (0 < {\mathrm{x}} \le 0.{4})$$
- (2) Intermetallic Phase Formation
  $${\mathrm{Li}}_{{\mathrm{x}}} {\mathrm{Ag}}\left( {\mathrm{s}} \right) + ({1} - {\mathrm{x}}){\text{ Li}}^{ + } + ({1} - {\mathrm{x}}){\text{ e}}^{ - } \to {\text{ LiAg }}\left( {\mathrm{s}} \right)$$
- (3) Li Plating
  $${\text{LiAg }}\left( {\mathrm{s}} \right) + {\mathrm{Li}}^{ + } + {\mathrm{e}}^{ - } \to {\text{ Li}}\left( {\mathrm{s}} \right) + {\mathrm{Ag}}\left( {\mathrm{s}} \right)$$
- (4) Dealloying Step
  7 $${\mathrm{Li}}\left( {\mathrm{s}} \right) \, \to {\text{ Li}}^{ + } + {\mathrm{e}}^{ - } \to {\text{ LiAg }}\left( {\mathrm{s}} \right) \, \to {\text{ Li}}_{{\mathrm{x}}} {\text{Ag }}\left( {\mathrm{s}} \right) \, \to {\text{ Ag }}\left( {\mathrm{s}} \right)$$

Figure 3a confirms that the resulting Li plating grows laterally atop the Ag–Li intermetallic layer. Therefore, rather than nonlocalized dendritic growth driven by Li self-diffusion barriers, the Ag–Li compound controlled Li deposition toward a planar morphology.

Surface chemical bonds were monitored using in situ Raman spectroscopy during cycling (2 mA cm^−2^, 1 mAh cm^−2^). In situ Raman measurements are taken on the surface of the Li (Fig. S37). The bare-Li cell exhibited peaks at approximately 540 cm^−1^ (Li–F) [59], 1200–1210 cm^−1^ (LiTFSI) [60], 1390 cm^−1^ (D band) [61], 1600–1750 cm^−1^ (NO_3_^−^) [62], 2061 cm^−1^ (C≡C) [66], and approximately 2300 cm^−1^ (*sp*^2^-C) [66] as shown in Fig. 4i. The BL-Li cell exhibited peaks at approximately 400 cm^−1^ (LiOH) [66], 900 cm^−1^ (Li_2_O_2_) [67], 1200–1210 cm^−1^ (LiTFSI) [60], 1390 cm^−1^ (D band) [61], 1600–1750 cm^−1^ (NO_3_^−^) [62], and 2061 cm^−1^ (C≡C) [66] as shown in Fig. 4j. The intensity ratios of LiTFSI (1200–1210 cm^−1^) [60] and NO_3_^−^ (1600–1750 cm^−1^) [62] peaks in the bare-Li vs. BL-Li cells were 1.28:1 and 1.59:1, respectively, indicating greater retention of electrolyte species on the bare electrode surface. Prolonged LiTFSI/LiNO_3_ coordination slowed Li^+^ desolvation and impeded interfacial kinetics. Therefore, the reduced electrolyte residue on BL–Li surfaces favored faster desolvation kinetics

### Electrochemical Performance of Full Cells

**Fig. 5:**
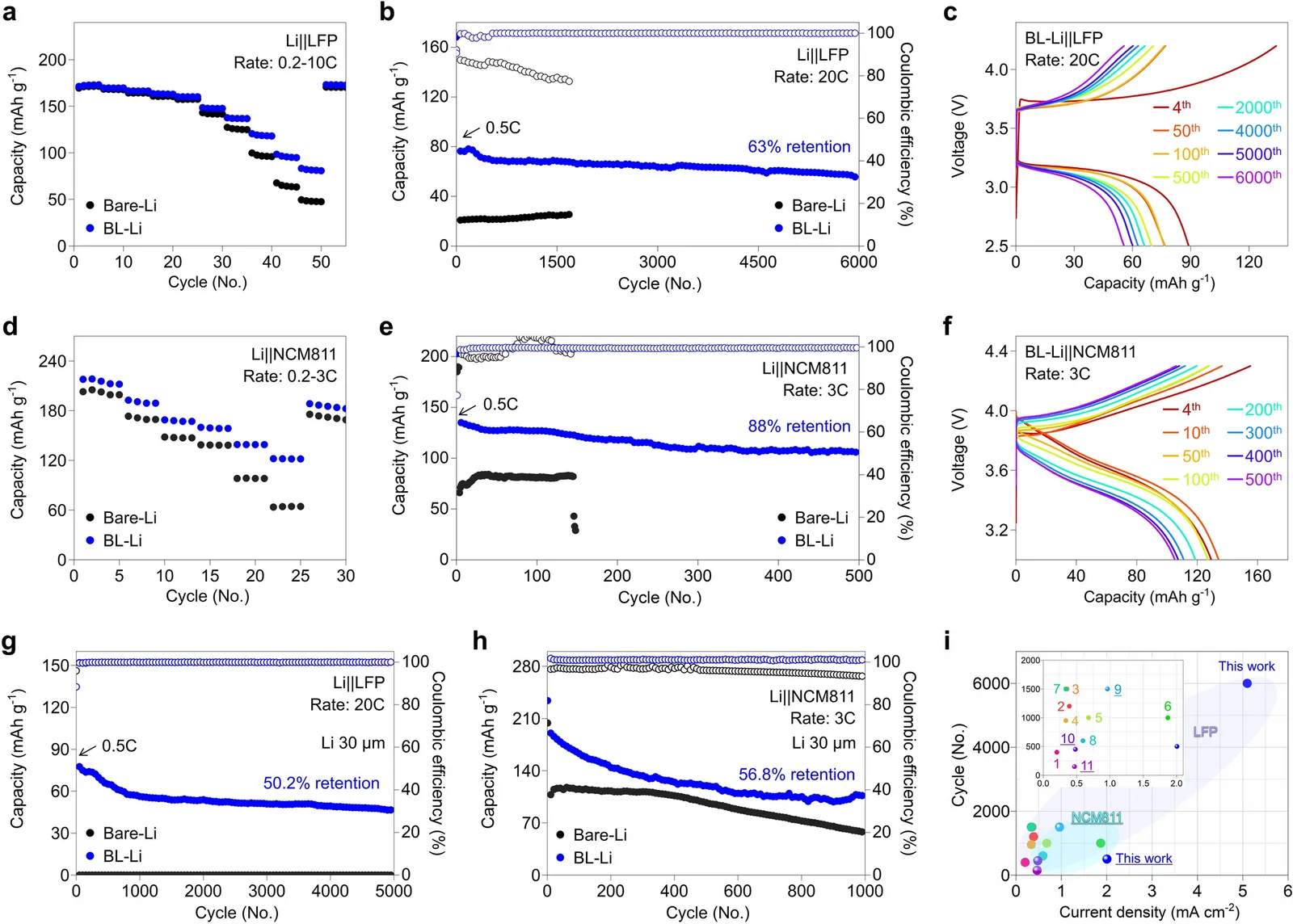
**a** Electrochemical performance of full cells. Rate performance, **b** cycling performance at 20 C, and **c** corresponding galvanostatic curves of bare-Li||LFP and BL-Li||LFP. **d** Rate performance and **e** cycling performance at 3 C and **f** corresponding galvanostatic curves of bare-Li||NCM811 and BL-Li||NCM811. Cycling performance at 20 C with thin Li anode with **g** LFP cathode and **h** NCM811 cathode. **i** Comparison diagram of electrochemical performance.

LFP and NCM811 full cells were assembled to probe kinetic behavior at varying C-rates. At C-rates of 0.2, 0.4, 0.6, 0.8, 1, 2, 3, 5, 8, and 10 C, the bare-Li‖LFP cell exhibited specific capacities of 170, 168, 164, 160, 157, 141, 125, 96, 63, and 48 mAh g^−1^ (returning to 170 mAh g^−1^ at 0.2 C), whereas the BL-Li‖LFP cell achieved specific capacities of 170, 169, 166, 163, 160, 147, 136, 118, 95, and 81 mAh g^−1^ (also recovering to 170 mAh g^−1^ at 0.2 C). Notably, at 10 C the BL-Li‖LFP cell’s capacity was nearly double than that of the bare-Li cell. Under an extreme 20 C cycling test, the bare-Li‖LFP cell’s capacity increased to only 20 mAh g^−1^, briefly increasing to 25 mAh g^−1^ before Coulombic efficiency collapsed. In contrast, the BL-Li‖LFP cell exhibited a capacity of 88 mAh g^−1^ and retained 63% of its capacity after 6000 cycles, with a voltage hysteresis of 0.576, 0.509, 0.522, 0.561, 0.547, 0.538, 0.575, and 0.584 V from cycle 4 to cycle 6000, demonstrating suppressed electrode degradation and stable impedance during prolonged high-rate operation. In analogous tests with NCM811 cathodes at 0.2, 0.4, 0.6, 1, 2, 3, and 0.2 C, the bare-Li‖NCM811 cell yielded 202, 171, 147, 138, 98, 64, and 178 mAh g^−1^, whereas the BL-Li‖NCM811 cell delivered 218, 190, 167, 159, 139, 121, and 188 mAh g^−1^, with more than a twofold higher capacity at 3 C. At 3 C cycling, the bare-Li‖NCM811 cell peaked late at 82 mAh g^−1^, but suffered unstable Coulombic efficiency and precipitous capacity fade by cycle 148. In contrast, the BL-Li‖NCM811 cell exhibited a capacity of 135 mAh g^−1^ and maintained 70.8% of its capacity over 6000 cycles, exhibiting voltage hysteresis of 0.390, 0.386, 0.427, 0.448, 0.561, 0.625, 0.656, and 0.659 V from cycle 4 to cycle 500. In addition, the galvanostatic charge–discharge profiles of the Li‖LFP and Li‖NCM811 cells operated at 0.2 C are presented in Fig. S38. The galvanostatic curves shown in Fig. 5c, f were obtained under high C-rate conditions and therefore exhibit relatively large voltage hysteresis between the charge and discharge processes. In contrast, the cells operated at a low C-rate of 0.2 C display lower and more stable voltage profiles. As the C-rate increases, overpotential during galvanostatic charge–discharge becomes larger due to combined ohmic, interfacial, and mass transport limitations, particularly under diffusion-limited conditions at high rates. Subsequently, performance is evaluated with high‐loading cathodes (Fig. S39). High‐loading electrodes, owing to their increased thickness, exhibited substantially higher *R*_ct_. In addition, reduced electronic conductivity increases the ohmic drop, and ion‐diffusion limitations further contribute to resistance. These factors collectively increased the overall overpotential of cell and hindered stable battery operation. For the bare‐Li‖8 mg cm^−2^ LFP cell, the initial discharge capacity of 139 mAh g^−1^ decreased to 112 mAh g^−1^ after 150 cycles, corresponding to a capacity retention of 80.6%. In contrast, the BL‐Li‖8 mg cm^−2^ LFP cell achieved an initial capacity of 155 mAh g^−1^ and retained 137 mAh g^−1^ after 150 cycles, achieving 88.4% retention. For the bare‐Li‖11 mg cm^−2^ NCM811 cell, the initial discharge capacity of 101 mAh g^−1^ decreased to 93 mAh g^−1^ after 80 cycles, corresponding to a capacity retention of 92%. In contrast, the BL‐Li‖11 mg cm^−2^ NCM811 cell achieved an initial capacity of 126 mAh g^−1^ and retained 122 mAh g^−1^ after 80 cycles, achieving 96.8% retention.

To probe Li^+^ electrochemistry at the electrode, we recorded cyclic voltammetry (CV) profiles at varying scan rates. The BL–Li‖LFP full cell exhibited steeper CV peaks and increased integrated currents, reflecting enhanced Li^+^ diffusion. At 0.1 mV s^−1^, the Fe^2+^/Fe^3+^ redox peak separation decreases from 0.31 V in bare-Li‖LFP to 0.20 V in BL–Li‖LFP, highlighting that the BL–Li anode stabilizes and reverses the LFP cathode more effectively (Fig. S40). Similarly, the BL–Li‖NCM811 cell exhibits sharper and broader peaks (Fig. S41), with the Ni^2+^/Ni^3+^ separation decreasing to 0.08 vs. 0.11 V in bare-Li‖NCM811, confirming accelerated interfacial kinetics. The dI/dV values were extracted from the linear regions of the CV current–voltage curves. In the anodic region of the LFP cells, the bare-Li‖LFP cell exhibited a conductance of 34.5 mA V^−1^, whereas the BL-Li‖LFP cell exhibited a conductance of 56.34 mA. In the cathodic region, the values were −36.9 and −40.1 mA, respectively (Fig. S40c). For the NCM811 cells, the anodic d*I*/d*V* values for bare-Li‖NCM811 and BL-Li‖NCM811 cells were 51.9 and 97.1 mA, respectively, and the cathodic values were −37.4 and −73.5 mA, respectively (Fig. S41c). For a given voltage change Δ*V*, a larger d*I*/d*V* indicated larger current change ΔI flow, corresponding to a lower *R*_ct_ (Δ*V*/Δ*I*). Therefore, the use of BL–Li anode reduced overpotential and accelerated reaction kinetics compared to the bare-Li anode. Measured Li⁺ diffusion coefficients (*D*_Li+_) further confirm this improvement: For LFP cells, *D*_Li+_ increased from 2.34 × 10^−8^ cm^2^ s^−1^ with bare-Li to 1.10 × 10^−7^ cm^2^ s^−1^ with BL–Li and for NCM811 cells from 8.65 × 10^−9^ to 1.66 × 10^−8^ cm^2^ s^−1^ under the same modification.

To investigate the effect of internal resistance of the cell during a single cycle, we performed a full charge–discharge cycle at 0.2 C and conducted EIS measurements at several discrete voltage points throughout that cycle (Fig. S42). The corresponding EIS fitting results are summarized in Tables S1 and S2. In the LFP and NCM811 full cells, the use of the BL–Li anode led to a substantial decrease in *R*ₒₕₘ. The *R*ₛₑᵢ values remained stable, confirming that the SEI layer was well-stabilized. In the bare-Li full cells, *R*_ct_ increased drastically at high state-of-charge, whereas in the BL-Li full cells it remained at a steady level of only tens of ohms, demonstrating excellent kinetic performance even at high charge/discharge rates and after thousands of cycles. Therefore, the BL-Li anode suppressed or stabilized all of the internal resistance components (ohmic, SEI, and charge transfer), effectively preventing performance degradation of the Li metal anode under extreme high-rate and long-term cycling. This finding makes it a highly promising strategy for designing high-energy–density and high-power LMBs.

Finally, full‐cell tests are carried out using an ultra‐thin (30 μm) Li metal anode (Fig. S43). As the anode thickness decreases, its sheet resistance (*R*_s_) increases proportionally as described in Eq. 8:

8 $$R_{s} = \rho /t$$

where *ρ* is the bulk resistivity and *t* is the electrode thickness. Under a constant charge–discharge current (I), the in‐plane potential drop within the electrode increases in direct proportion to *R*_s_:

9 $$\Delta V = I \times R_{{\mathrm{s}}}$$

This larger ΔV amplified non‐uniformity in the electrode’s potential distribution, creating steeper potential gradients across its surface. These gradients biased the current toward regions of lower local resistance, leading to localized current density “hotspots”. At these hotspots, the intensified electric field further accelerated Li‐ion reduction and dendritic growth. Consequently, a reduction in the Li anode thickness not only increases R_s_ but also exacerbates current localization and promotes dendrite formation.

The bare-30-µm Li‖LFP cell failed to exhibit any capacity, whereas the BL-30-µm Li‖LFP cell exhibited capacity of 92 mAh g^−1^, with 50.2% retention over 5000 cycles. Similarly, the bare-30-µm Li‖NCM811 cell fell from 107 to 57 mAh g^−1^ after 1000 cycles, whereas the BL-30-µm Li‖NCM811 cell retained 105 mAh g^−1^ of its initial capacity of 186 mAh g^−1^. Compared to previously reported full-cell performances of ASEI-based lithium batteries, our system demonstrates stable operation at higher current densities. In particularly, when paired with an LFP cathode, it achieved an extended cycle life of 6000 cycles, underscoring the exceptional performance of BL–Li (Fig. 5h and Table S3). These results confirmed that the bilayer ASEI promoted a broad current density distribution, lowered local current hotspots, suppressed resistance rise and electrode degradation, and achieved outstanding capability and cycle life even under harsh, ultrahigh-rate, and ultrathin-anode conditions.

## Conclusions

In this study, we demonstrate for the first time, a bilayer design fabricated via electrospinning. This bilayer was fabricated without additional bonding and consisted a fully electronic‐conductive layer and an ion‐conductive layer that did not transfer electrons. The fibrous structure, which facilitated electrolyte infiltration, lowered Li‐ion diffusion resistance and provided abundant active sites, enabling smooth spheroidal Li deposition. In addition, ample electron transport pathways homogenized local current density, preventing cell degradation and reducing *R*_ct_, further facilitating fast charge–discharge performance. The BL-Li anode maintained a low overpotential of approximately 120 mV for over 300 h under high current conditions (20 mA cm^−2^, 10 mAh cm^−2^) in Li symmetric‐cell tests. In full‐cell configurations with LFP and NCM811 cathodes, capacity retentions of 63% and 70.8% at 20 and 3 C were achieved, respectively. Furthermore, full‐cell tests conducted at the same high C‐rate using a thin Li anode, an environment prone to exacerbated potential‐distribution nonuniformity, demonstrated superior cyclability and capacity recovery. This promising bilayer approach for Li metal anodes provides a viable strategy for achieving high‐performance, high‐energy‐density Li metal batteries.

## Supplementary Information

Below is the link to the electronic supplementary material.

Supplementary file1 (DOCX 11671 KB)
