# Supplementary material for: Expediting Lithium Electrochemistry via a Bilayer for High-Rate Lithium Metal Batteries
Source: Nanomicro Lett. 2026 Mar 19;18:292. doi: 10.1007/s40820-026-02146-3 (PMC13000029; doi:10.1007/s40820-026-02146-3)
Supplement: Supplementary file 1 — Supplementary file1 (DOCX 11671 KB) [file 40820_2026_2146_MOESM1_ESM.docx]

Supporting Information for

**Expediting Lithium Electrochemistry via a Bilayer for High-Rate Lithium Metal Batteries**

Dongjoo Park^1^ and Dong-Wan Kim^1^*

^1^School of Civil, Environmental, and Architectural Engineering, Korea University, Seoul 02841, South Korea

*Corresponding author. E-mail: [dwkim1@korea.ac.kr](mailto:dwkim1@korea.ac.kr) (Dong-Wan Kim)

**Supplementary Table and Figures**

**
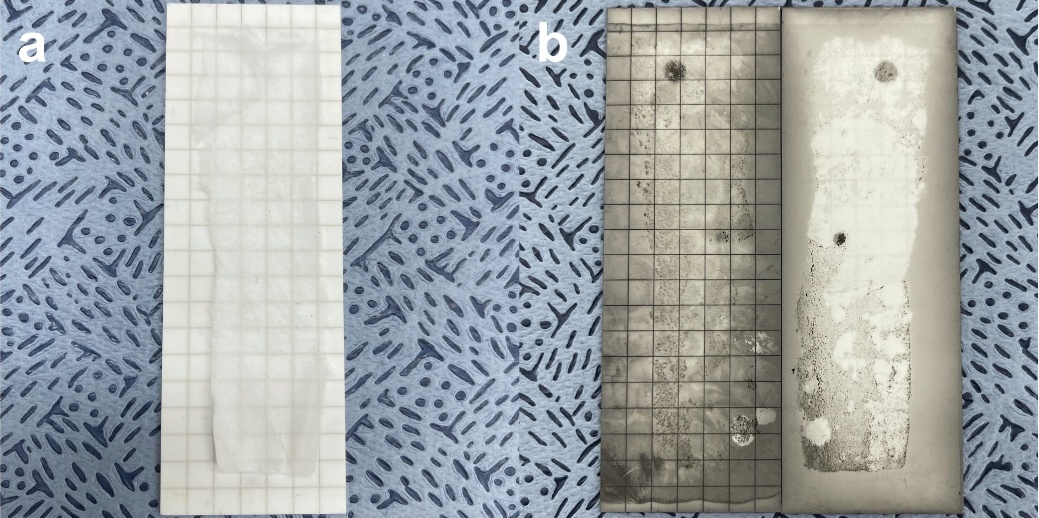
**

**Fig. S1** Photo images of L-layer before heat treatment (**a**) and after heat treatment (**b**)


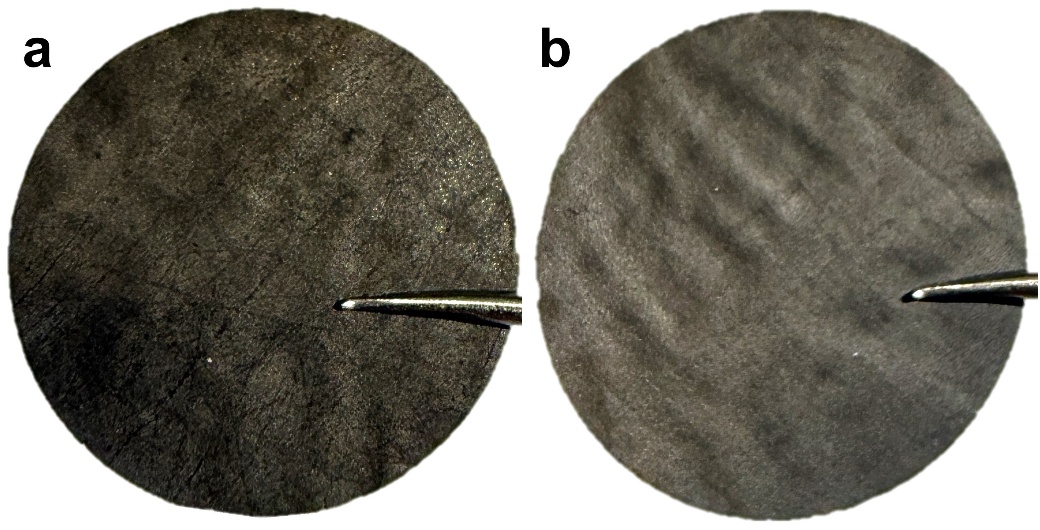


**Fig. S2** Photo images of BL-layer, L-layer (**a**) and C-layer (**b**)

**
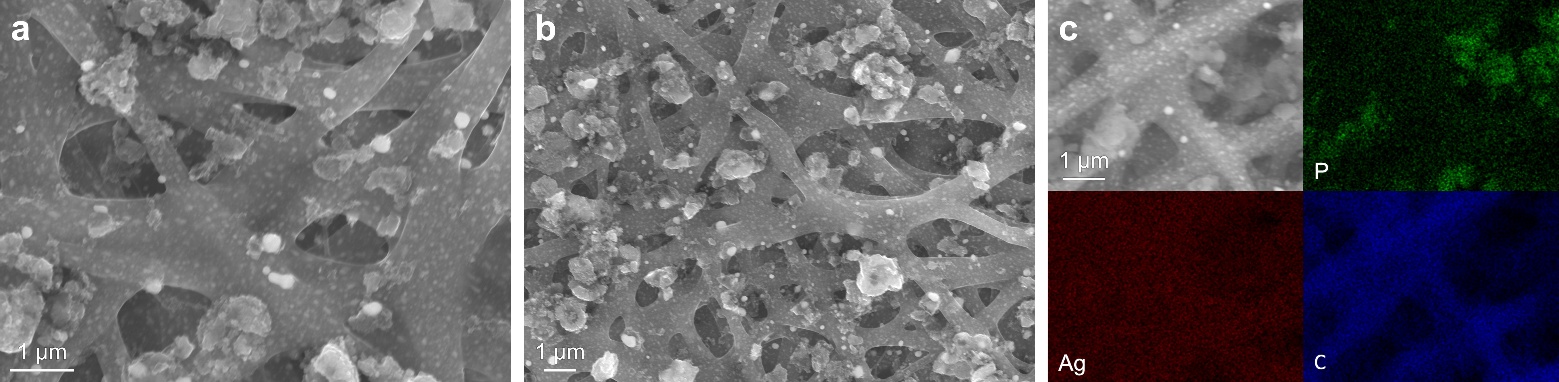
**

**Fig. S3** SEM images (**a, b**) and EDAX images (**c**) of L-layer

**
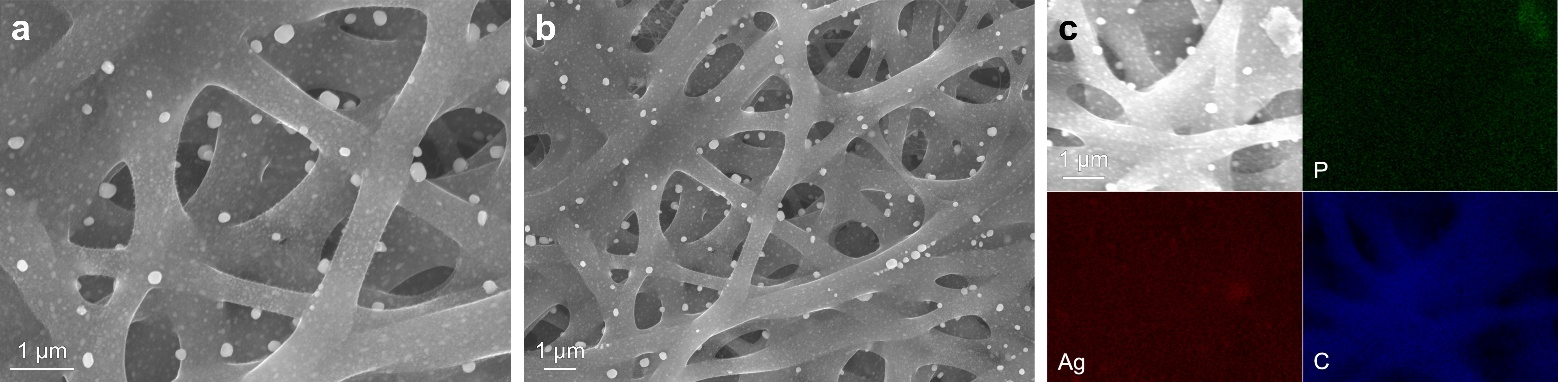
**

**Fig. S4** SEM images (**a, b**) and EDAX images (**c**) of C-layer

**
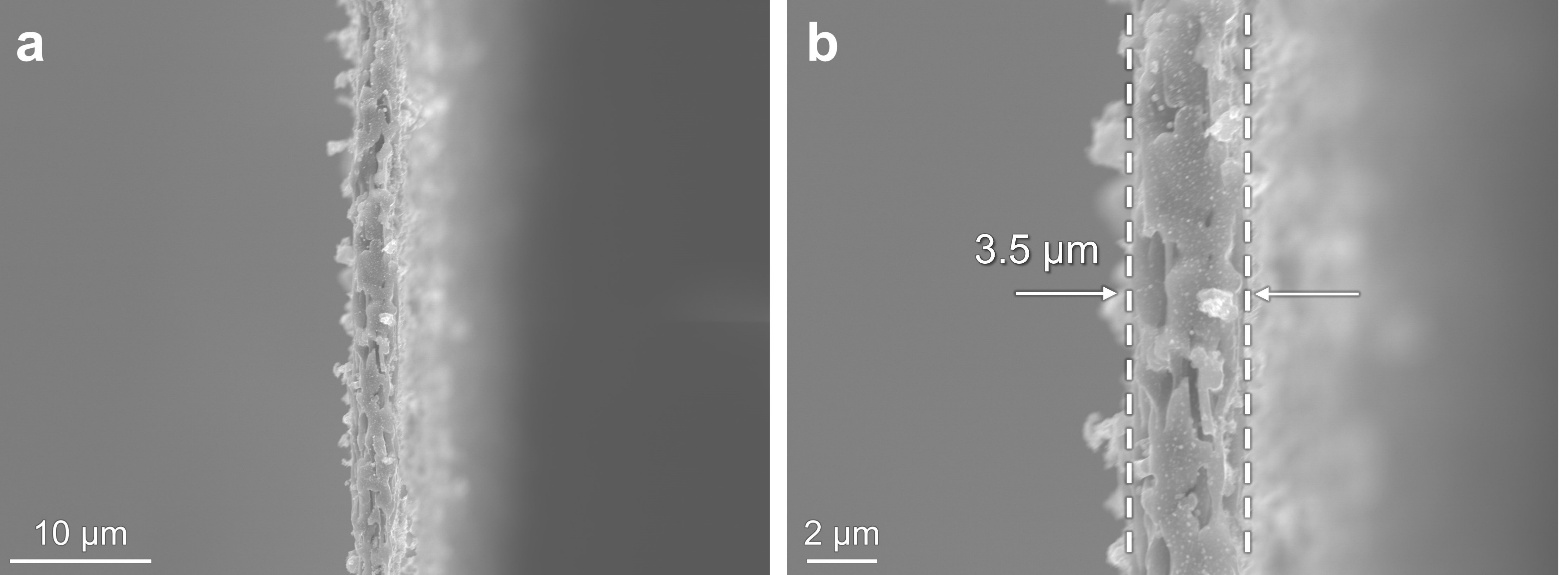
**

**Fig. S5** SEM Cross-sectional image of BL thickness

**
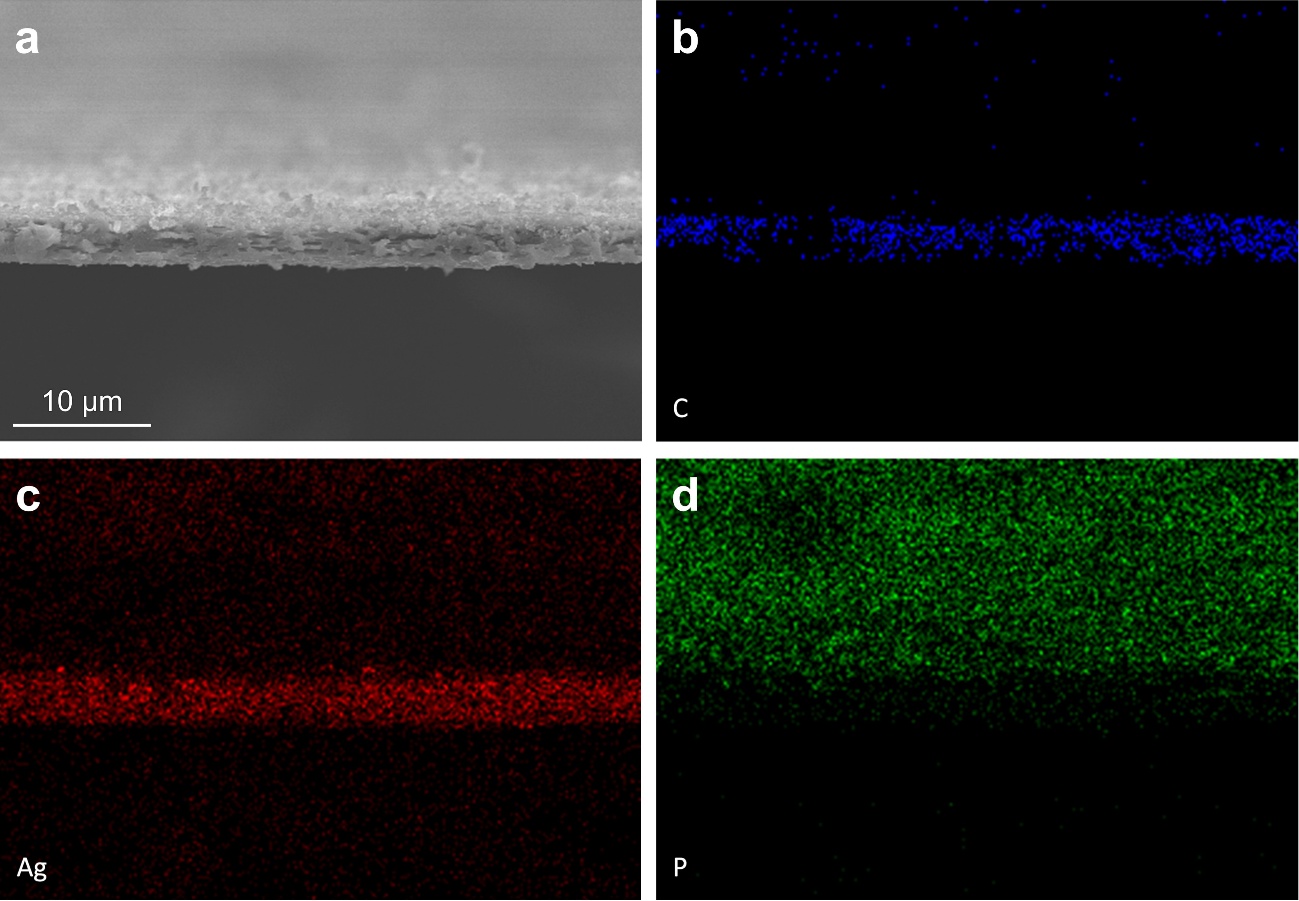
**

**Fig. S6** Cross-sectional SEM image of BL (**a**) and corresponding EDS elemental maps of C (**b**), Ag (**c**), and P (**d**)

**
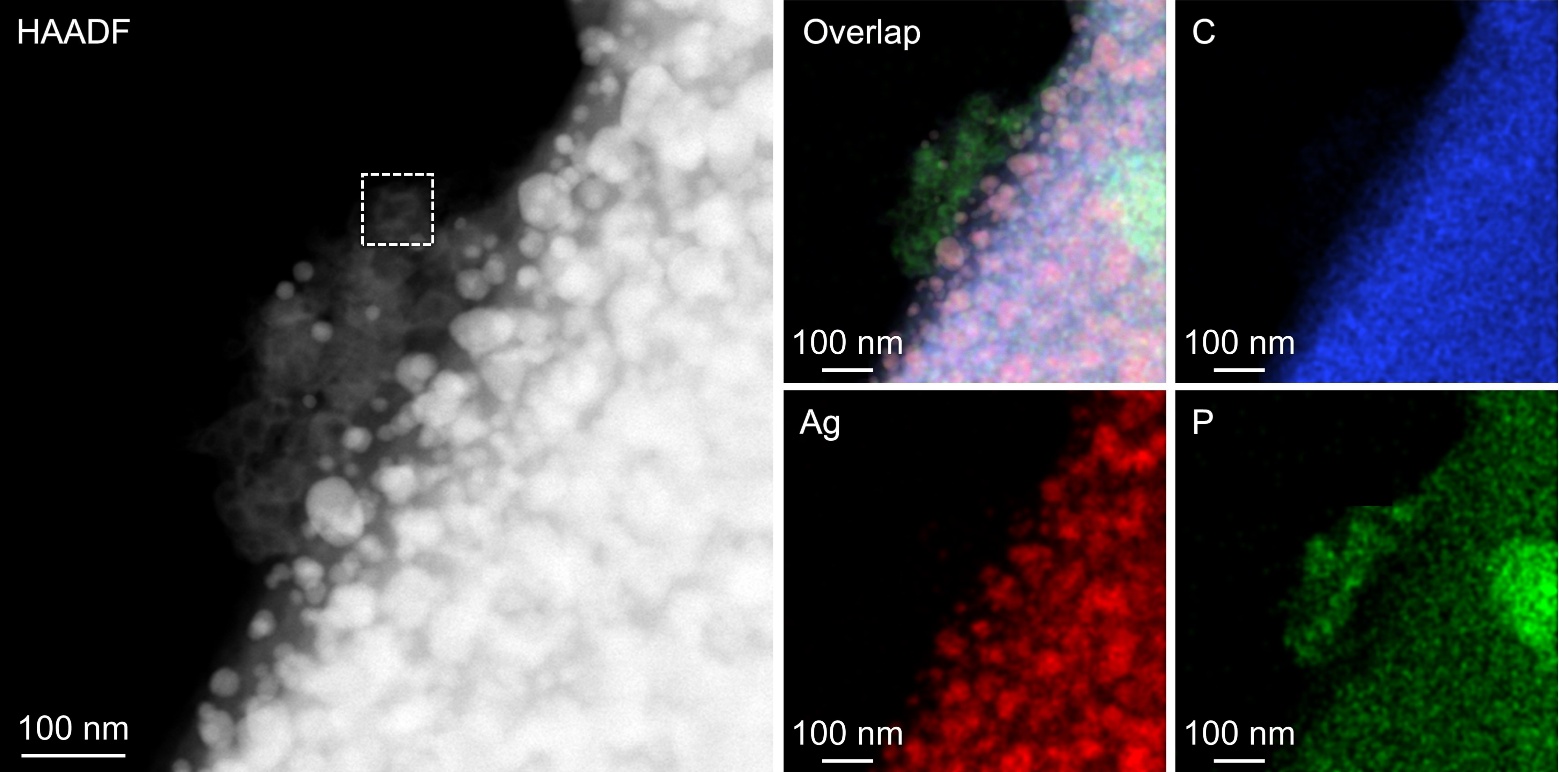
**

**Fig. S7** HAADF-STEM images of BL and corresponding elemental mapping

**
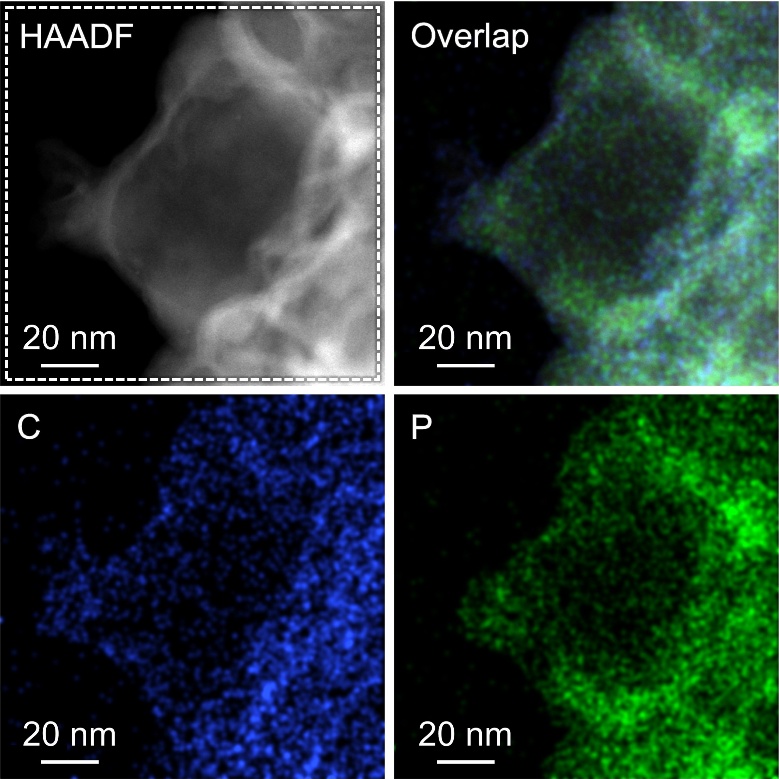
**

**Fig. S8** HAADF-STEM images of surface agglomerates on the BL (noted in Fig.Sx) and corresponding elemental mapping

**
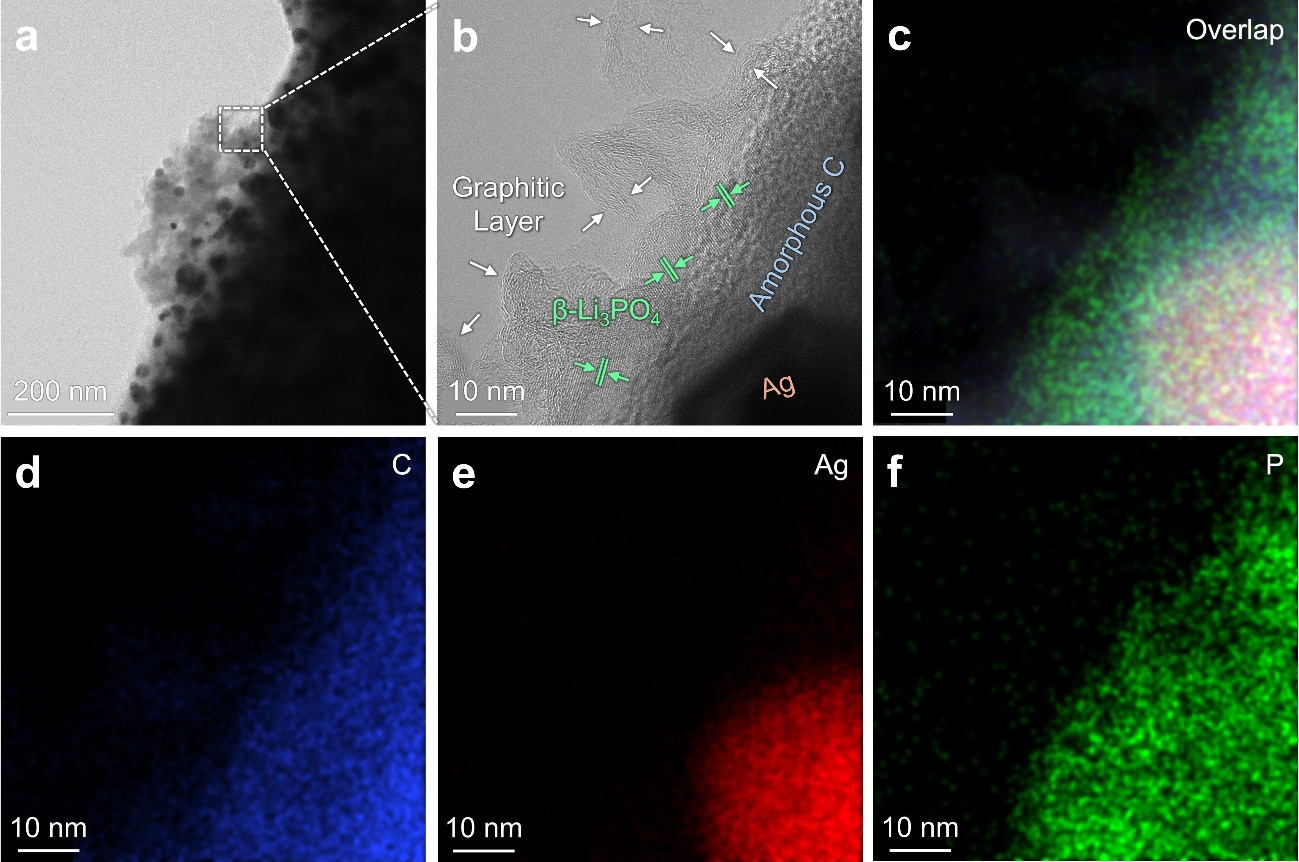
**

**Fig. S9** HAADF-STEM images of BL (**a**), surface agglomerates on the BL (noted in Fig.S9(a)) (**b**) and corresponding elemental mapping of overlapping image (**c**), C (**d**), Ag (**e**), and P (**f**)

**
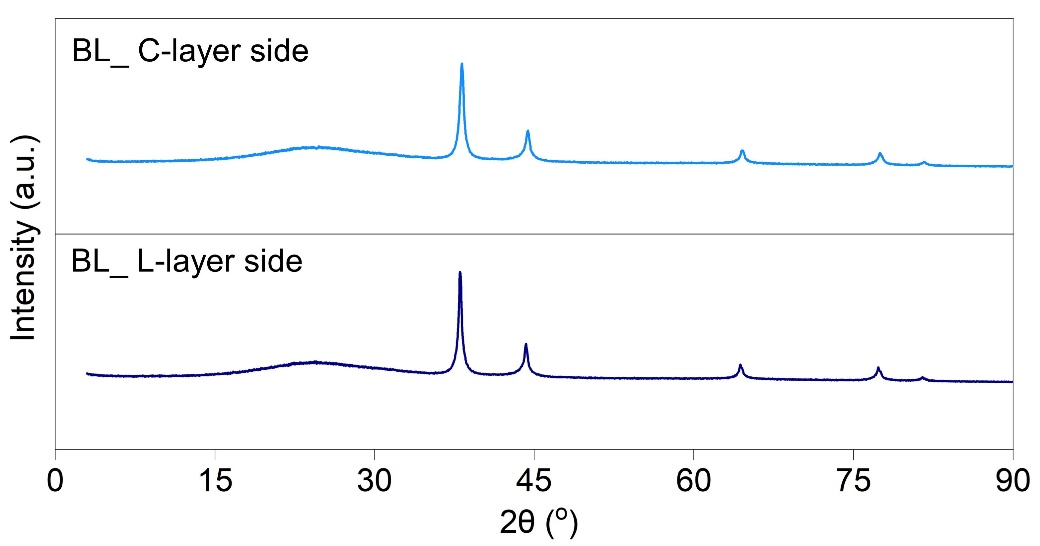
**

**Fig. S10** XRD spectra of BL, C-layer side (upper) and L-layer (lower)

**
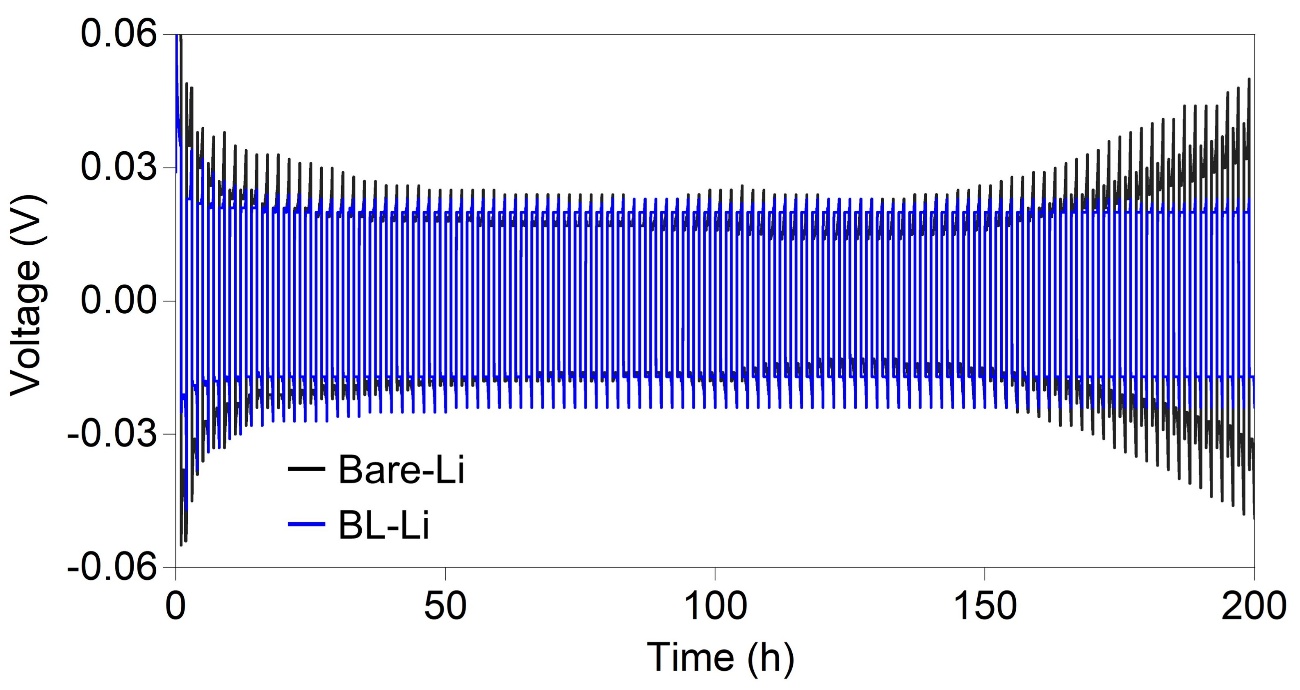
**

**Fig. S11** Li-Li symmetric cell under operating conditions of 1 mA cm⁻² and 1 mAh cm⁻² for 100 cycles

**
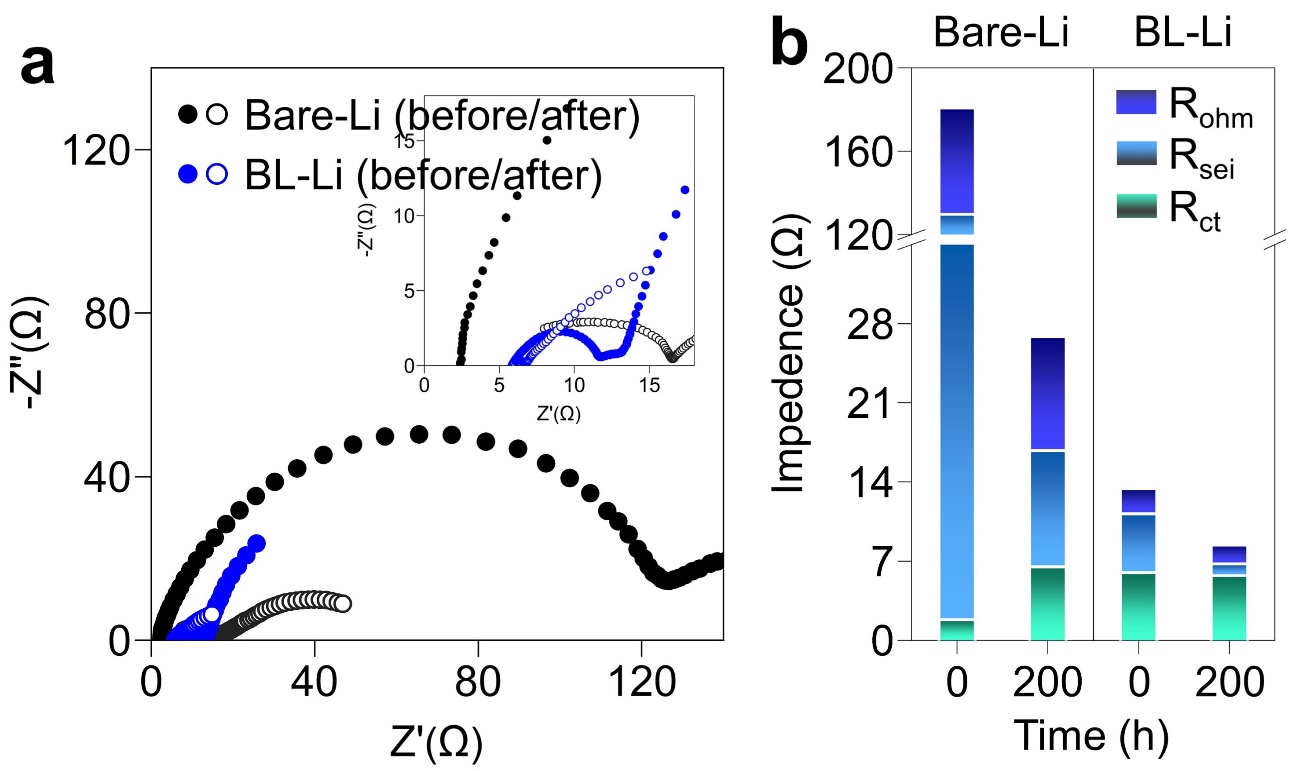
**

**Fig. S12** Comparison of interfacial resistance and overall kinetics before and after at 1 mA cm^-2^, 1 mAh cm^-2^ for 100 cycles

**
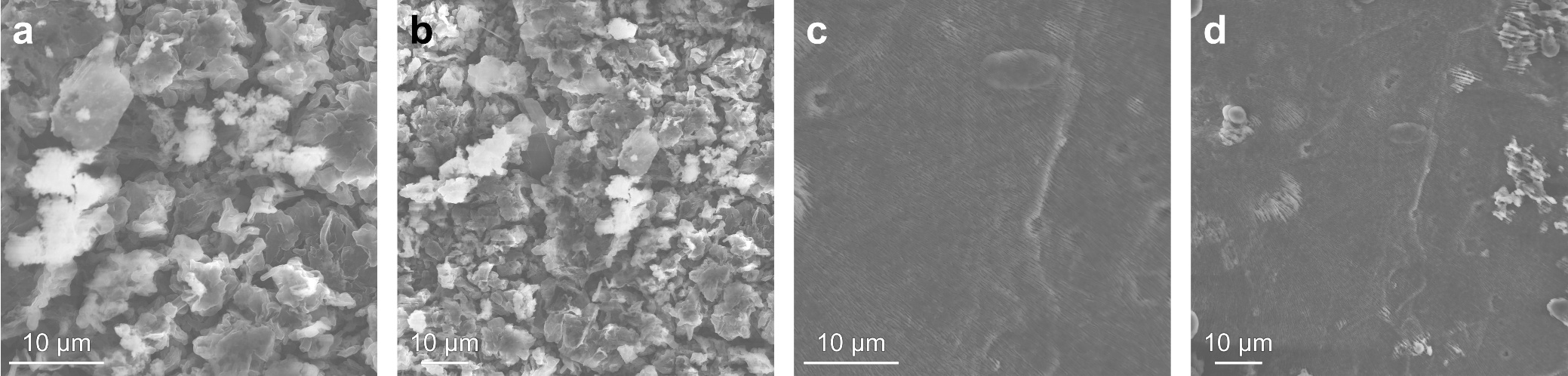
**

**Fig. S13** SEM Top view of after the Li-Li symmetric cells with operating conditions at 1 mA cm^−2^, 1 mAh cm^−2^ for 100 cycles of Bare-Li (**a, b**) and BL-Li (**c, d**)


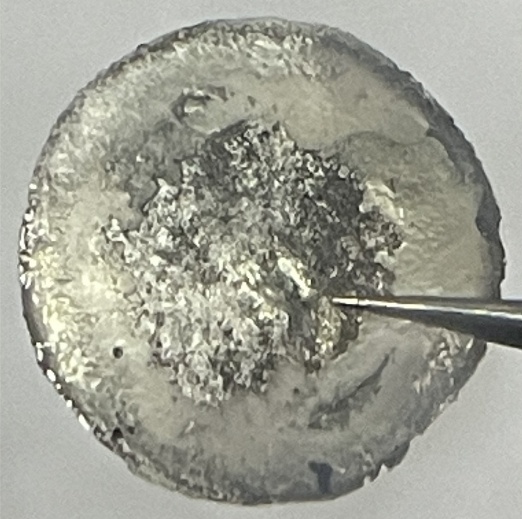


**Fig. S14** Photo image of Bare-Li after 100 cycles at 1 mA cm^−2^ with a fixed capacity of 1 mAh cm^−2^


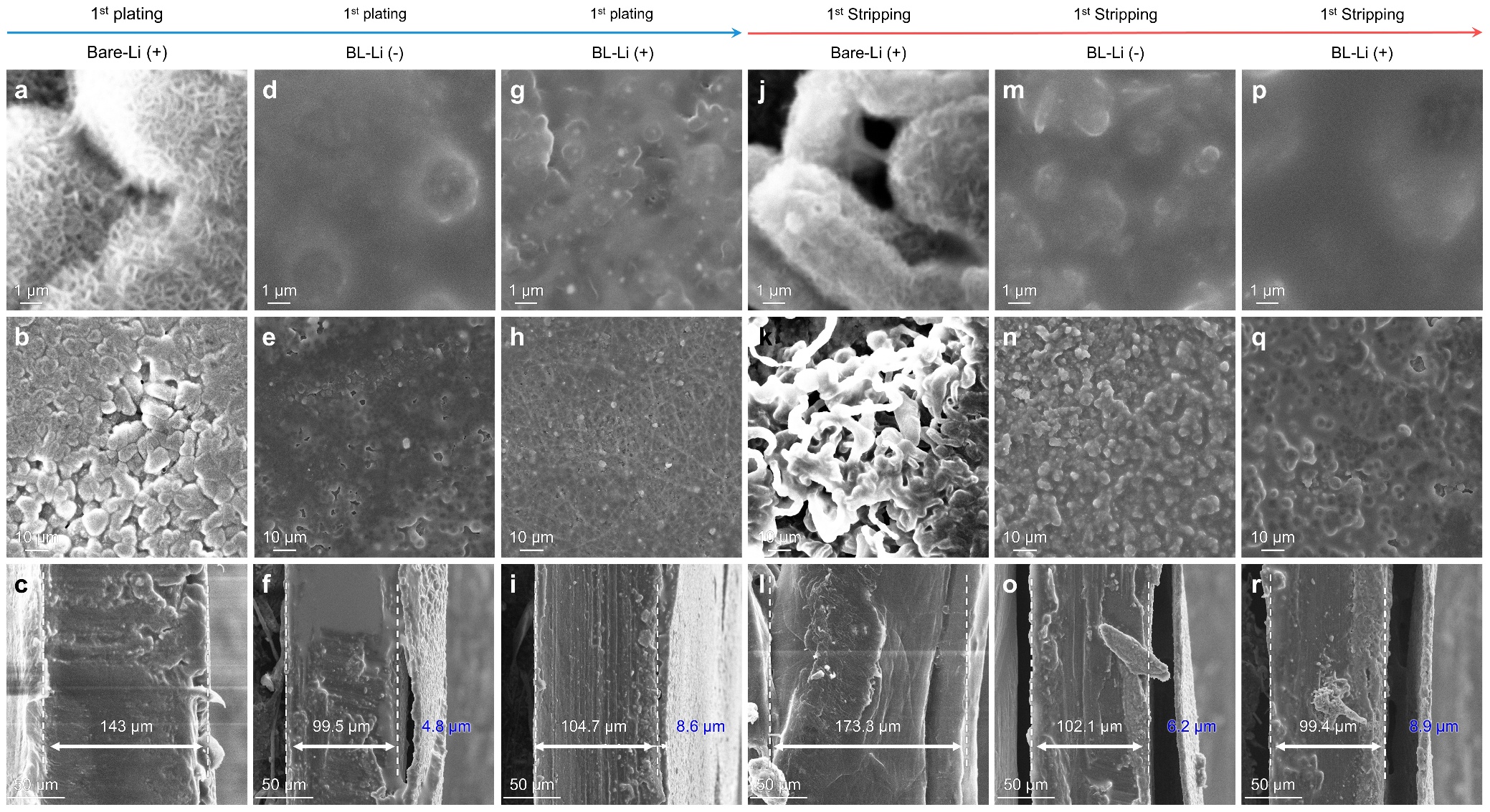


**Fig. S15** SEM plane/cross view images


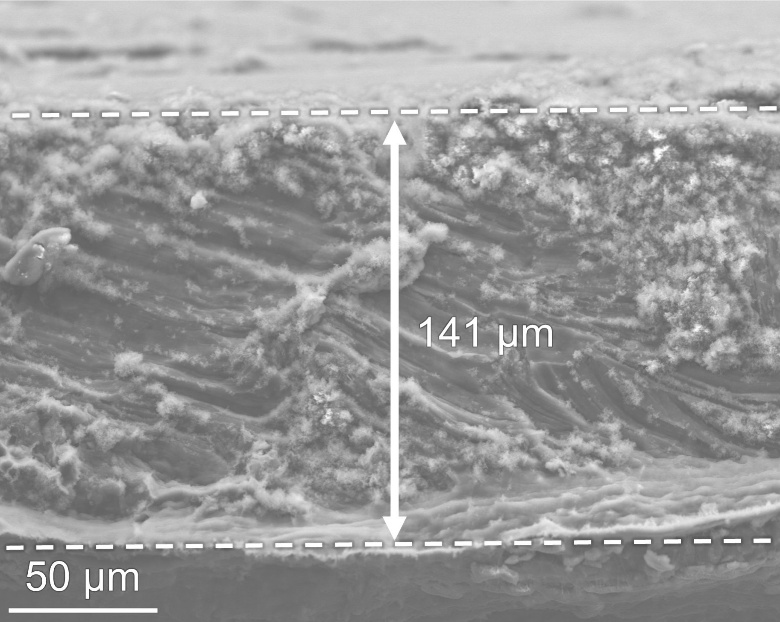


**Fig. S16** SEM cross view image of fresh-Li for comparison with Fig. S9


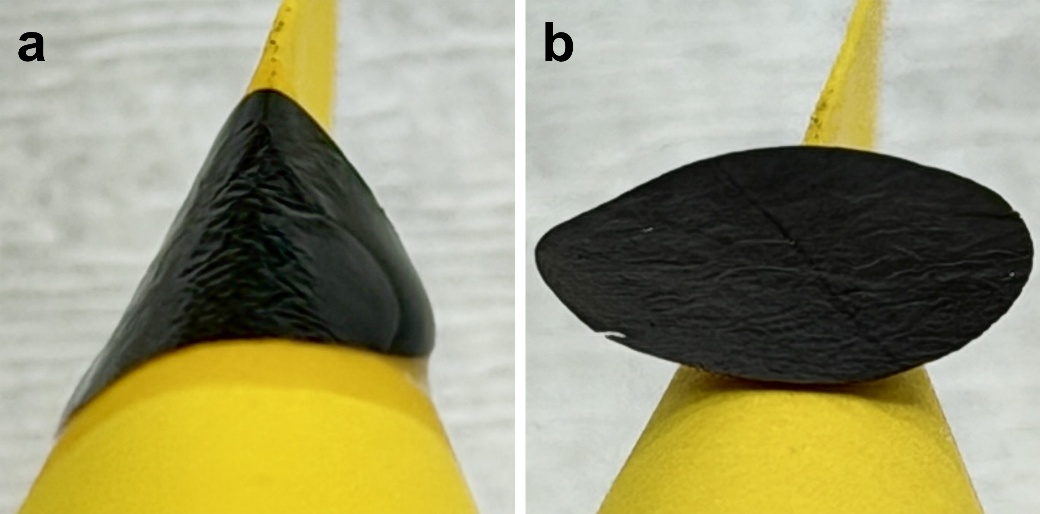


**Fig. S17** Optical photographs showing the BL in the wetted (**a**) and dry (**b**) states on a bending paper substrate


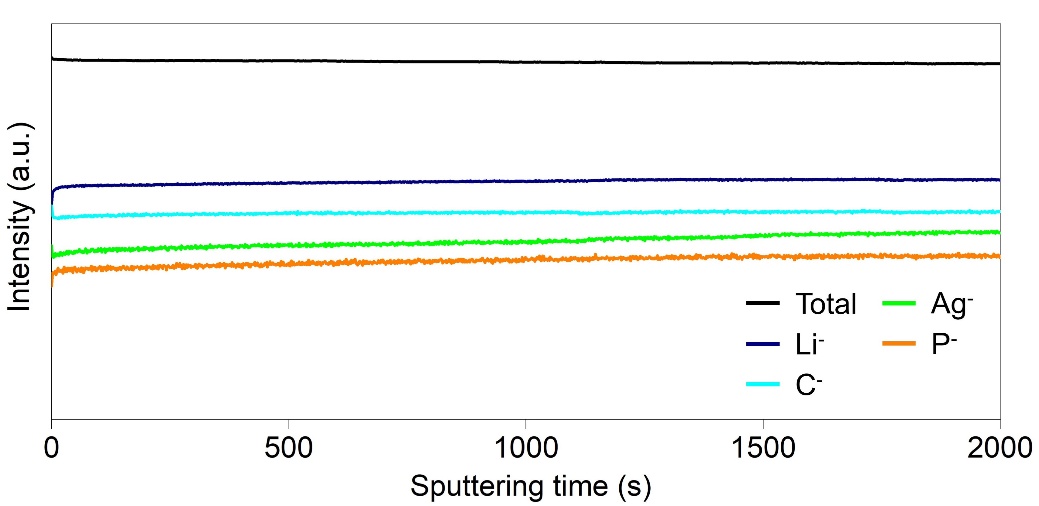


**Fig. S18** TOF-SIMS depth profiling of BL-Li cell under operating conditions of 1 mA cm⁻² and 1 mAh cm⁻² for 100 cycles

**
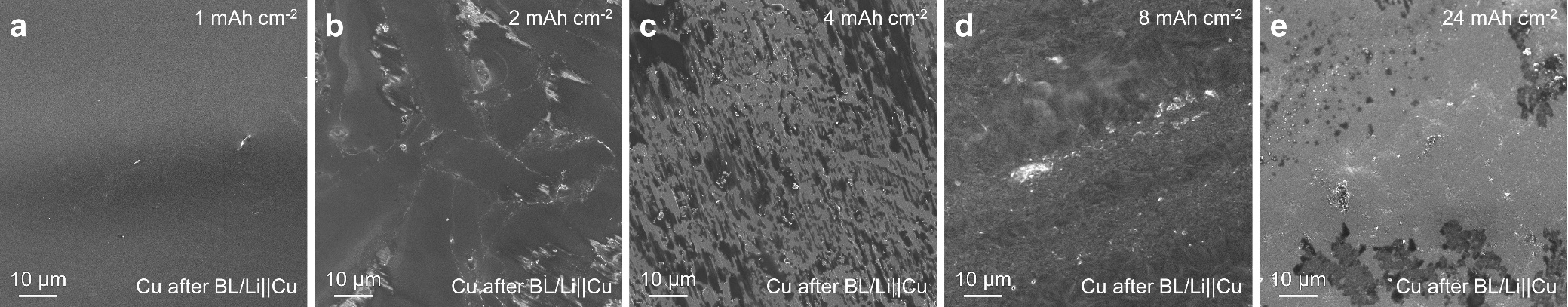
**

**Fig. S19** SEM images of Cu after asymmetric Cu-Li cells under operating conditions of 1, 2, 4, 8, and 24 mAh cm^−2^

**
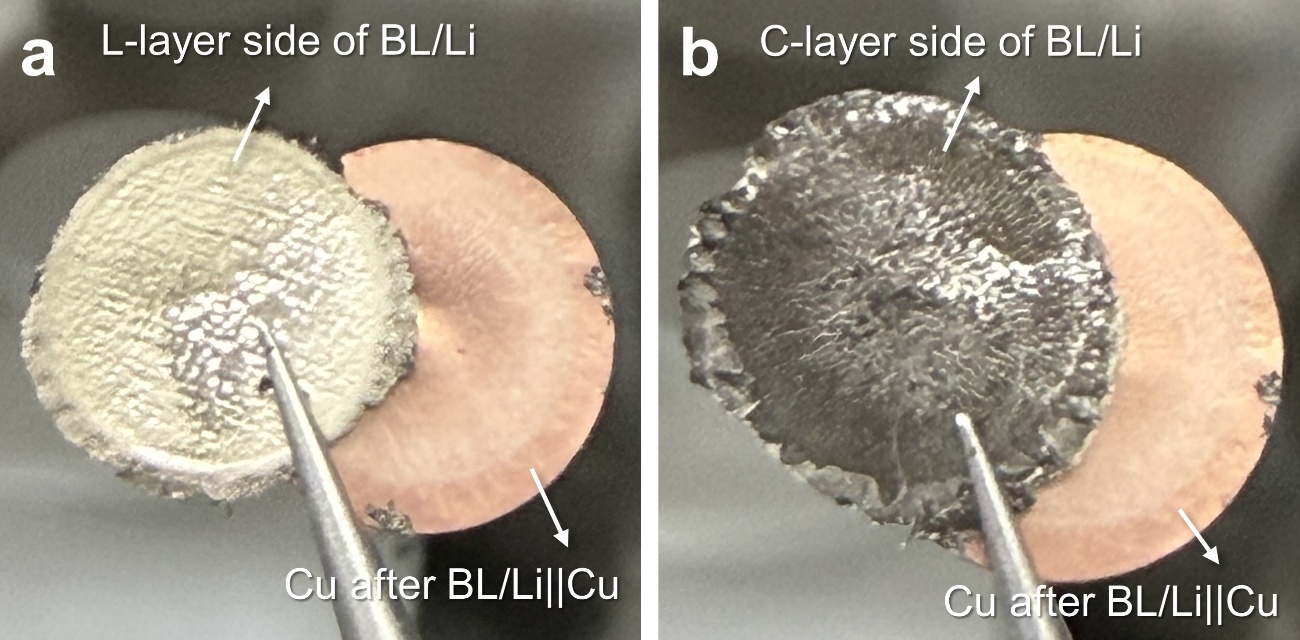
**

**Fig. S20** Photo images of asymmetric Cu-Li cells under operating conditions of 1 mA cm^−2^ for 24 h. L-layer side of BL/Li (**a**) and C-layer side of BL/Li (**b**)

**
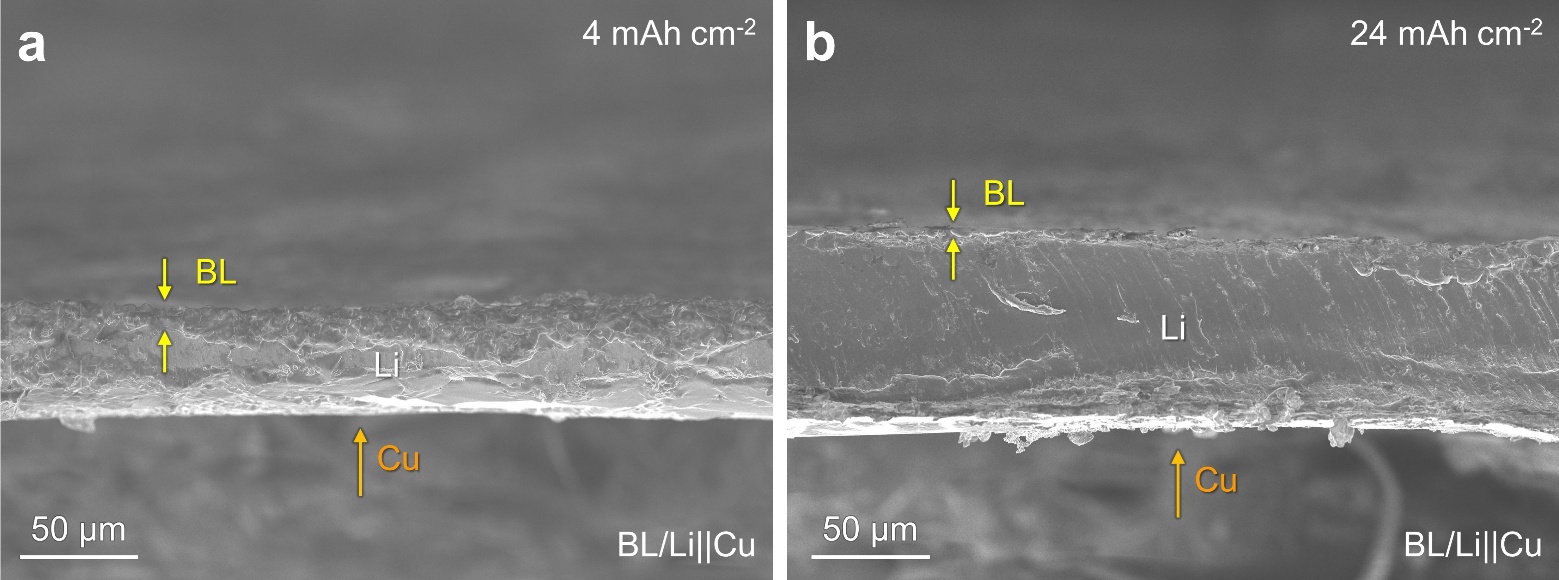
**

**Fig. S21** SEM Cross-sectional image after asymmetric Cu-Li cells under operating conditions of 4 mAh cm^−2^ (**a**) and 24 mAh cm^−2^ (**b**)

**
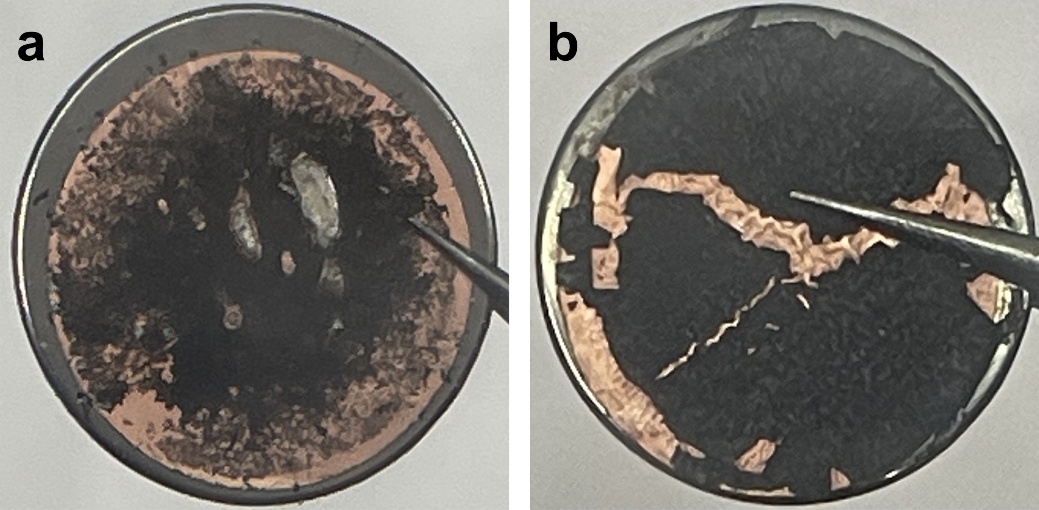
**

**Fig. S22** Cu electrode after 24 mAh cm^-2^ plating and 24 mAh cm^-2^ stripping

**
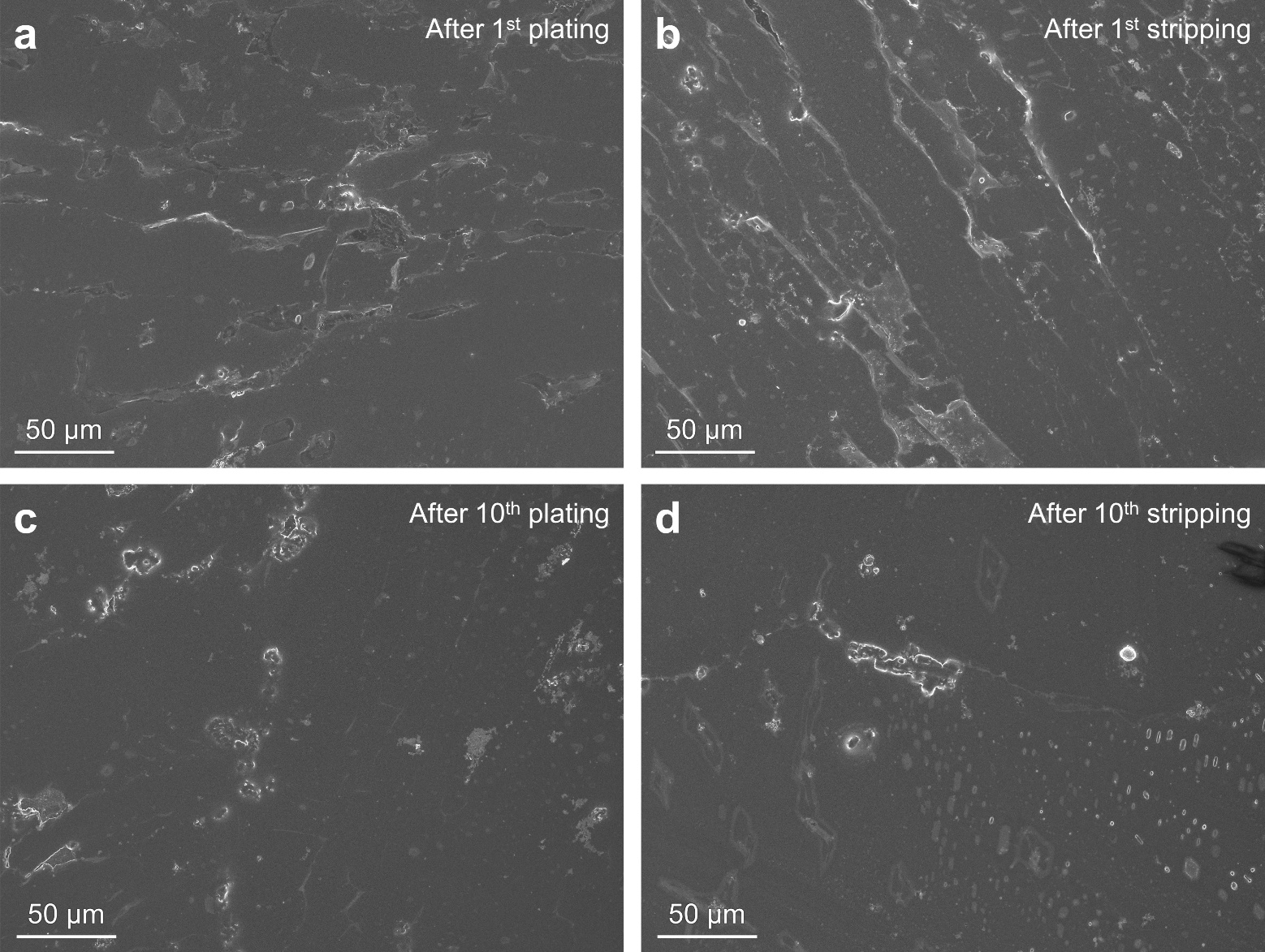
**

**Fig. S23** SEM image of Li after symmetric BL/Li cells under operating conditions of 1 mA cm^−2^ at 1 mAh cm^−2^ after 1^st^ plating (**a**), after 1^st^ stripping (**b**), after 10^th^ plating (**c**), and after 10^th^ stripping (**d**)

**
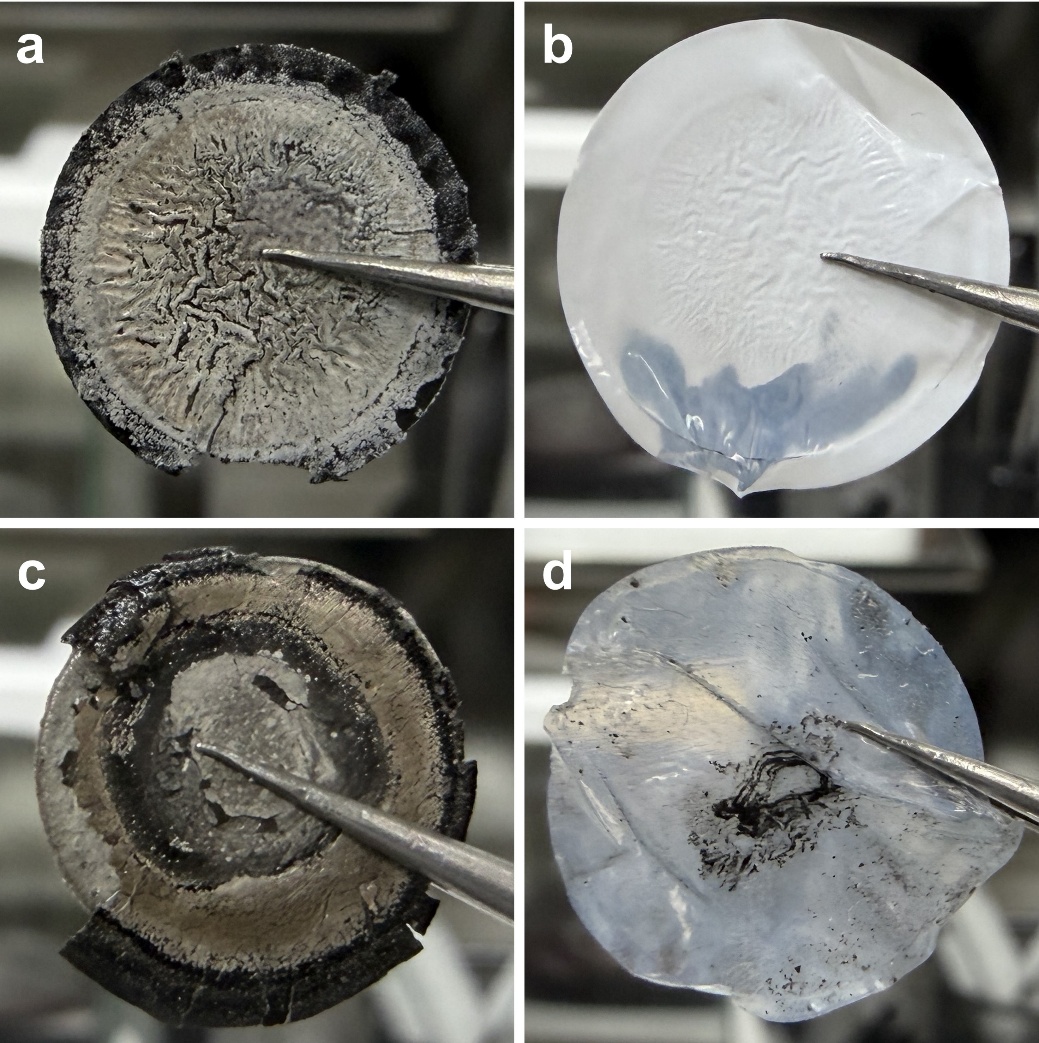
**

**Fig. S24** Optical images of BL/Li electrodes and separators after cell disassembly following Li plating/stripping at 20 mA cm⁻² and 10 mAh cm⁻² for 50 h (**a and b**) and 320 h (**c and d**)

**
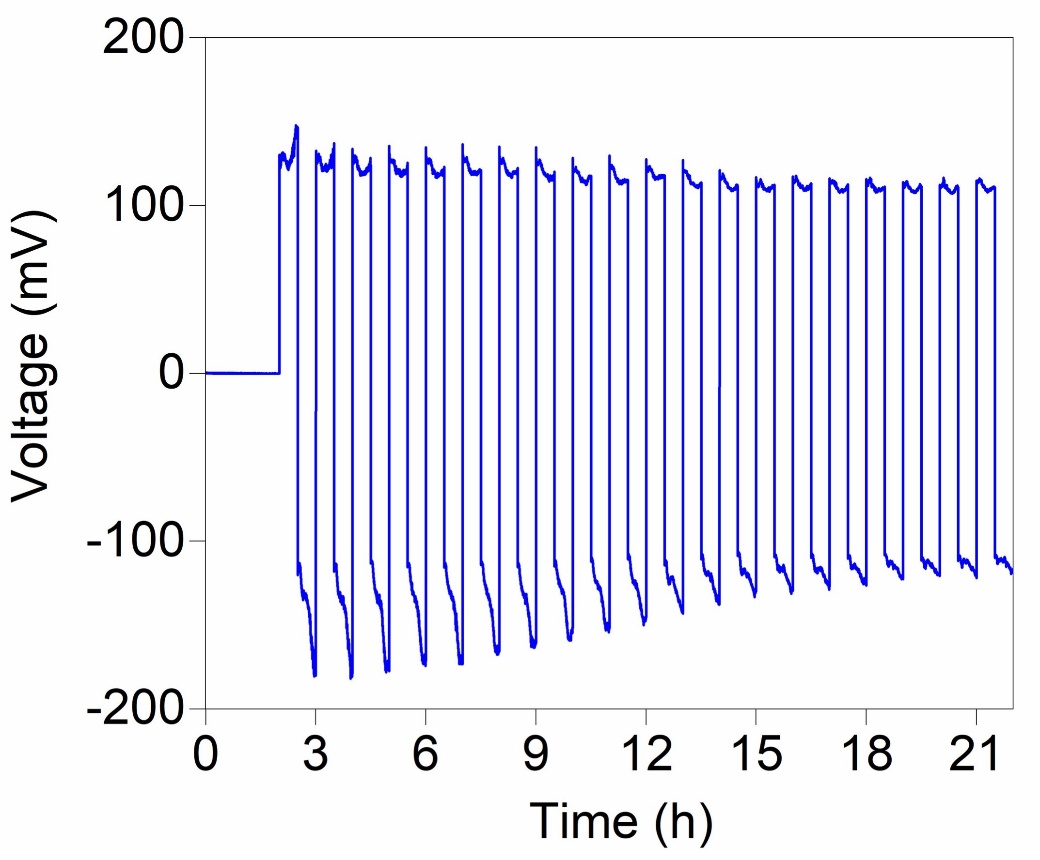
**

**Fig. S25** Galvanostatic charge–discharge voltage profiles of Li–Li symmetric cells with operating conditions at 20 mA cm^−2^, 10 mAh cm^−2^ for 20 h

**
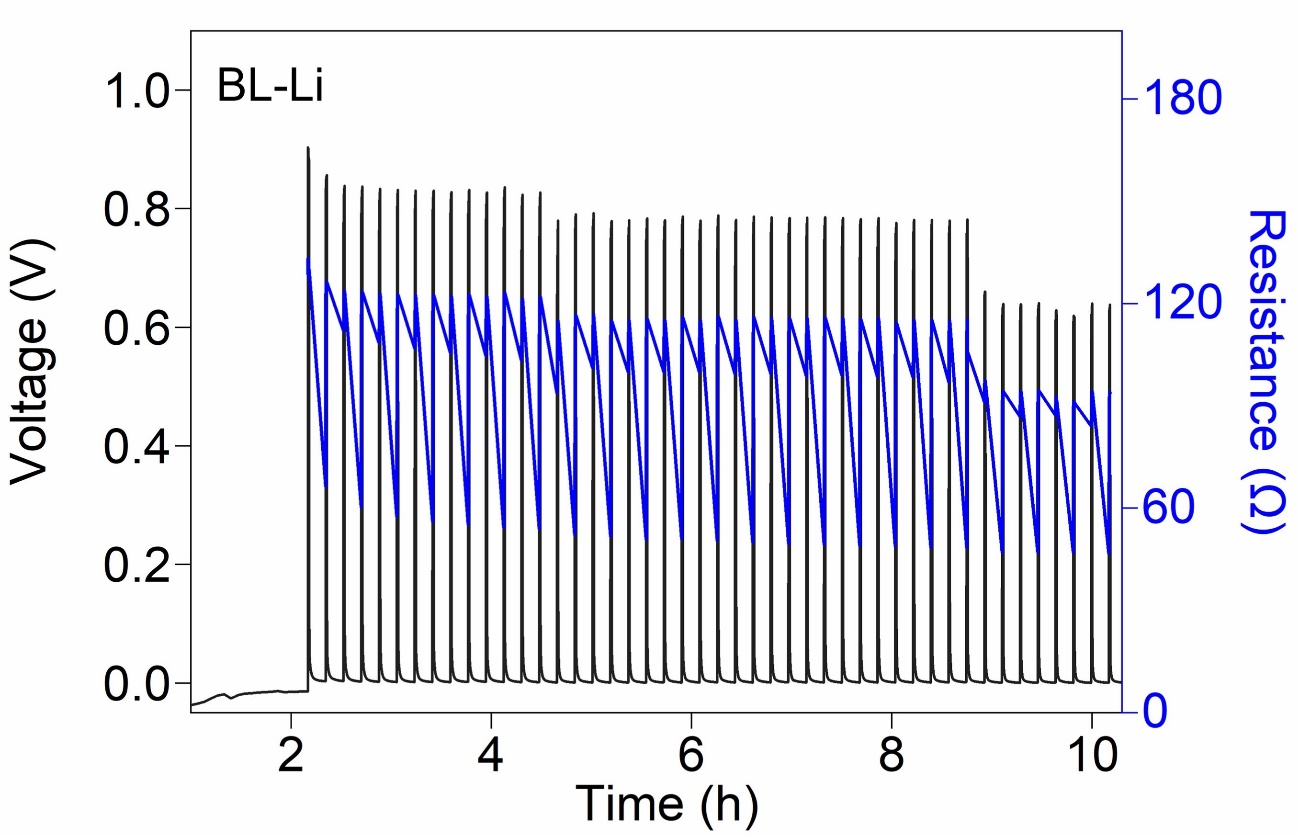
**

**Fig. S26** GITT profiles of a Li symmetric cell during lithium plating using the BL-Li at 20 mA cm⁻², reaching a cumulative areal capacity of 10 mAh cm⁻²

**
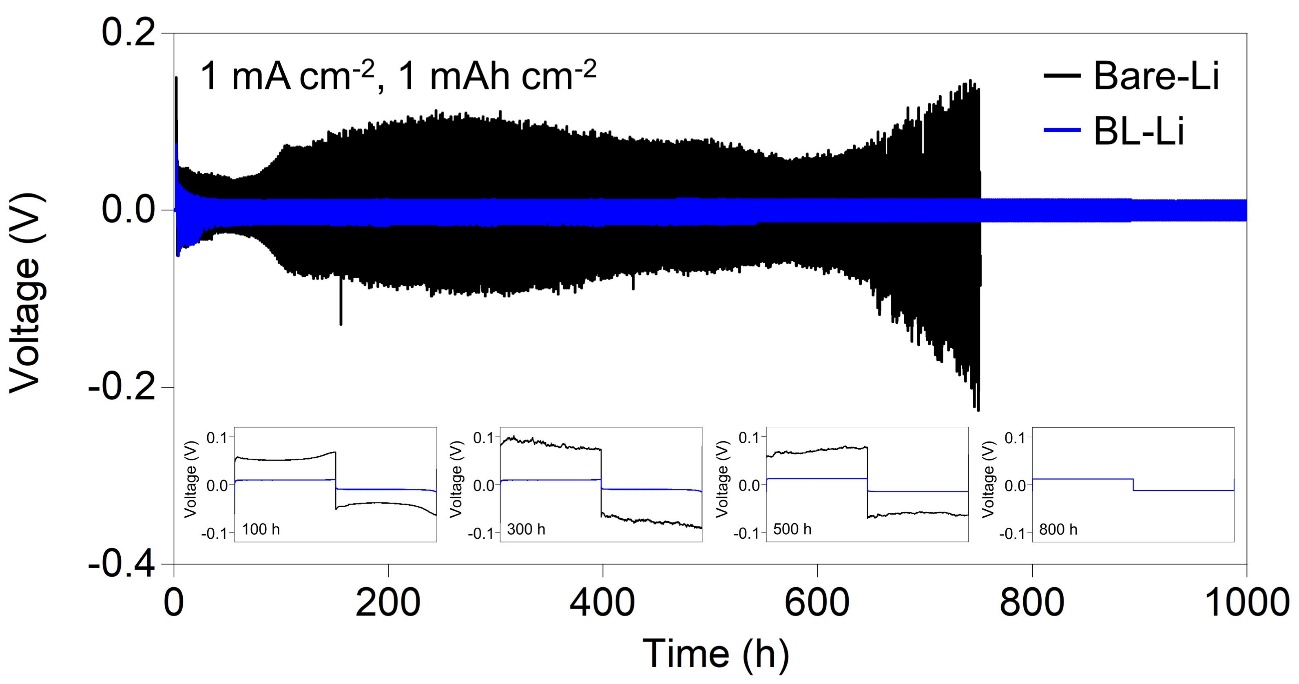
**

**Fig. S27** Galvanostatic charge-discharge voltage profiles of the Li-Li symmetric cells with operating conditions at 1 mA cm^−2^, 1 mAh cm^−2^ for 1000 h

**
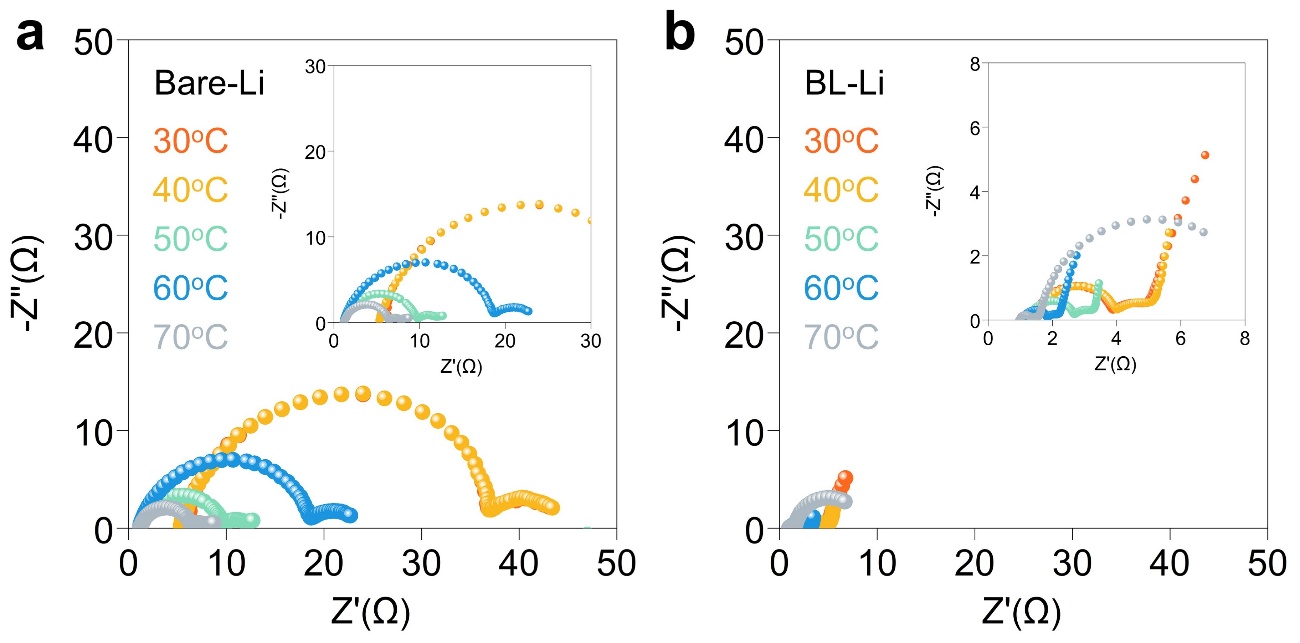
**

**Fig. S28** EIS measurement at different temperature (**a and b**)

**
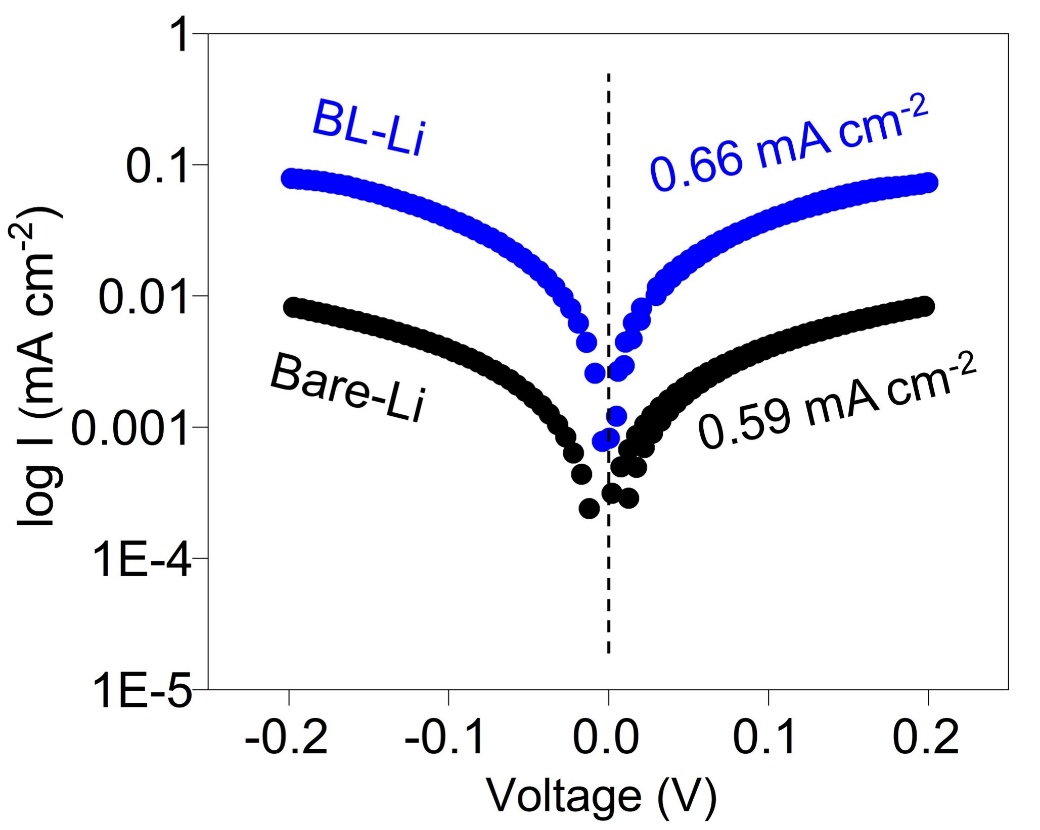
**

**Fig. S29** Tafel plots with scan rate 5 mV s^-1^ and calculated exchange-current densities (j_0_) in the Li symmetric cells

**
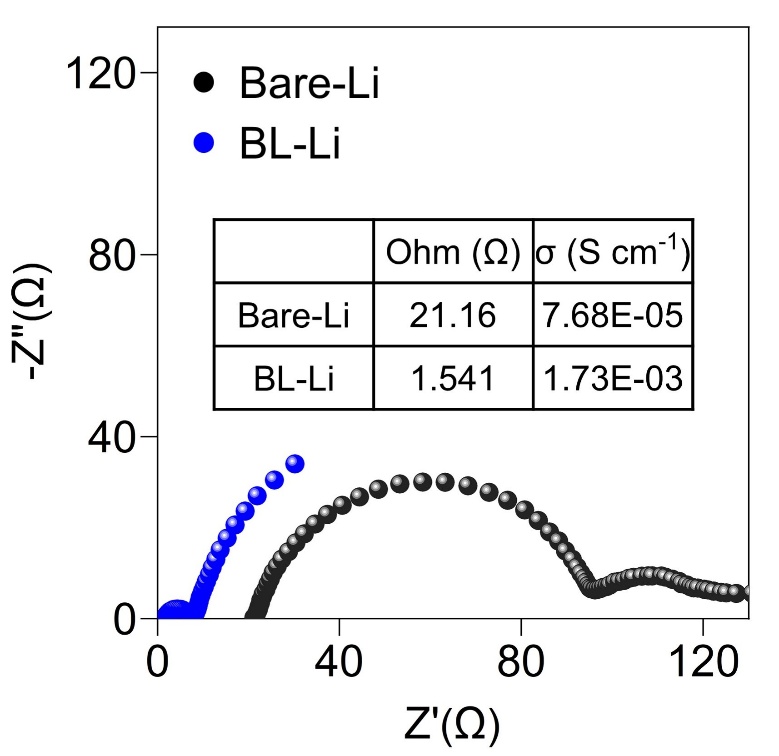
**

**Fig. S30** The Li^+^ ionic conductivity of Bare-Li and BL-Li

**
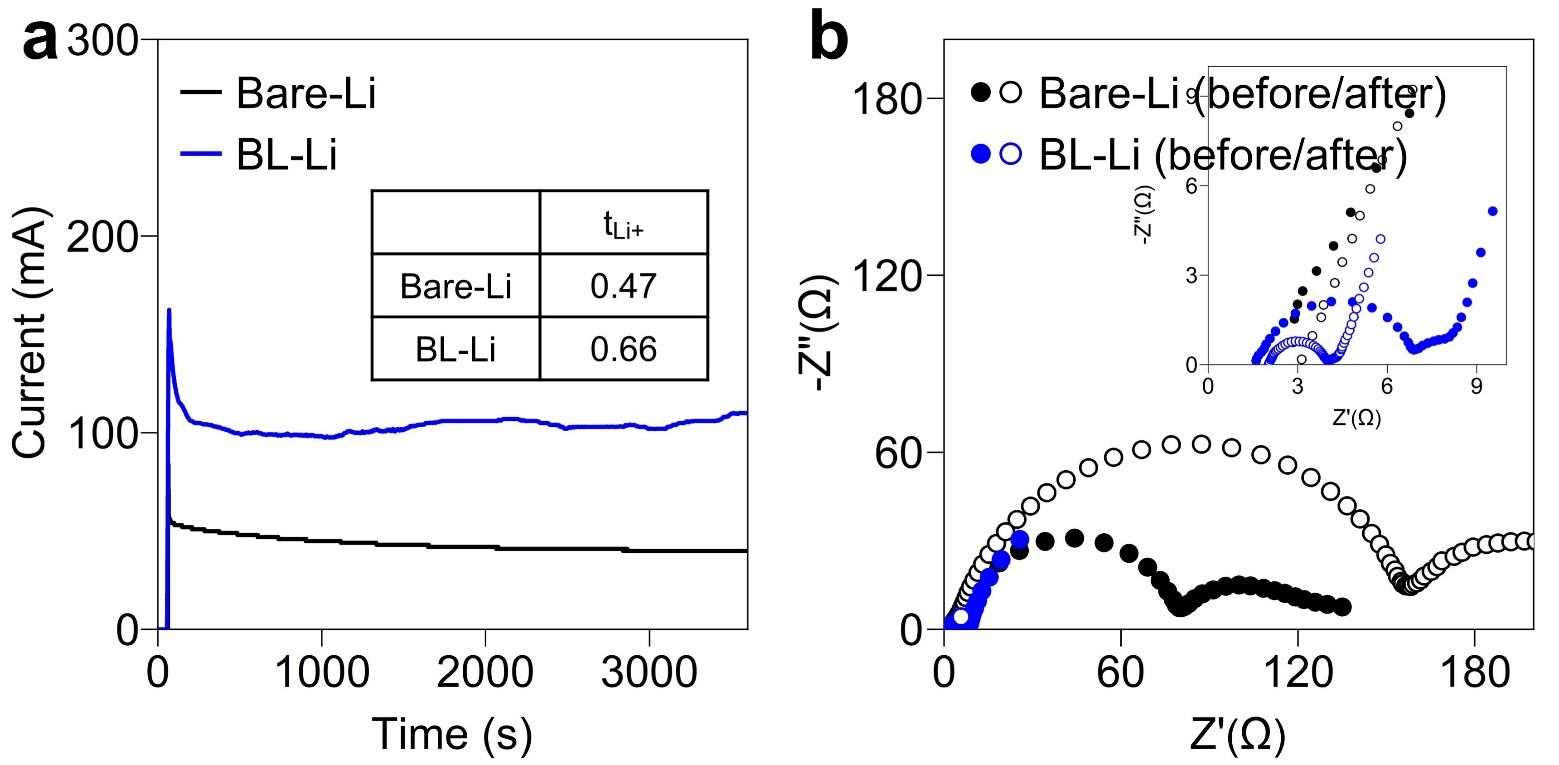
**

**Fig. S31** Chronoamperometry profile and transference number (**a**) and the EIS plots before and after chronoamperometry collected from a symmetric Bare-Li and BL-Li cells (**b**)

**
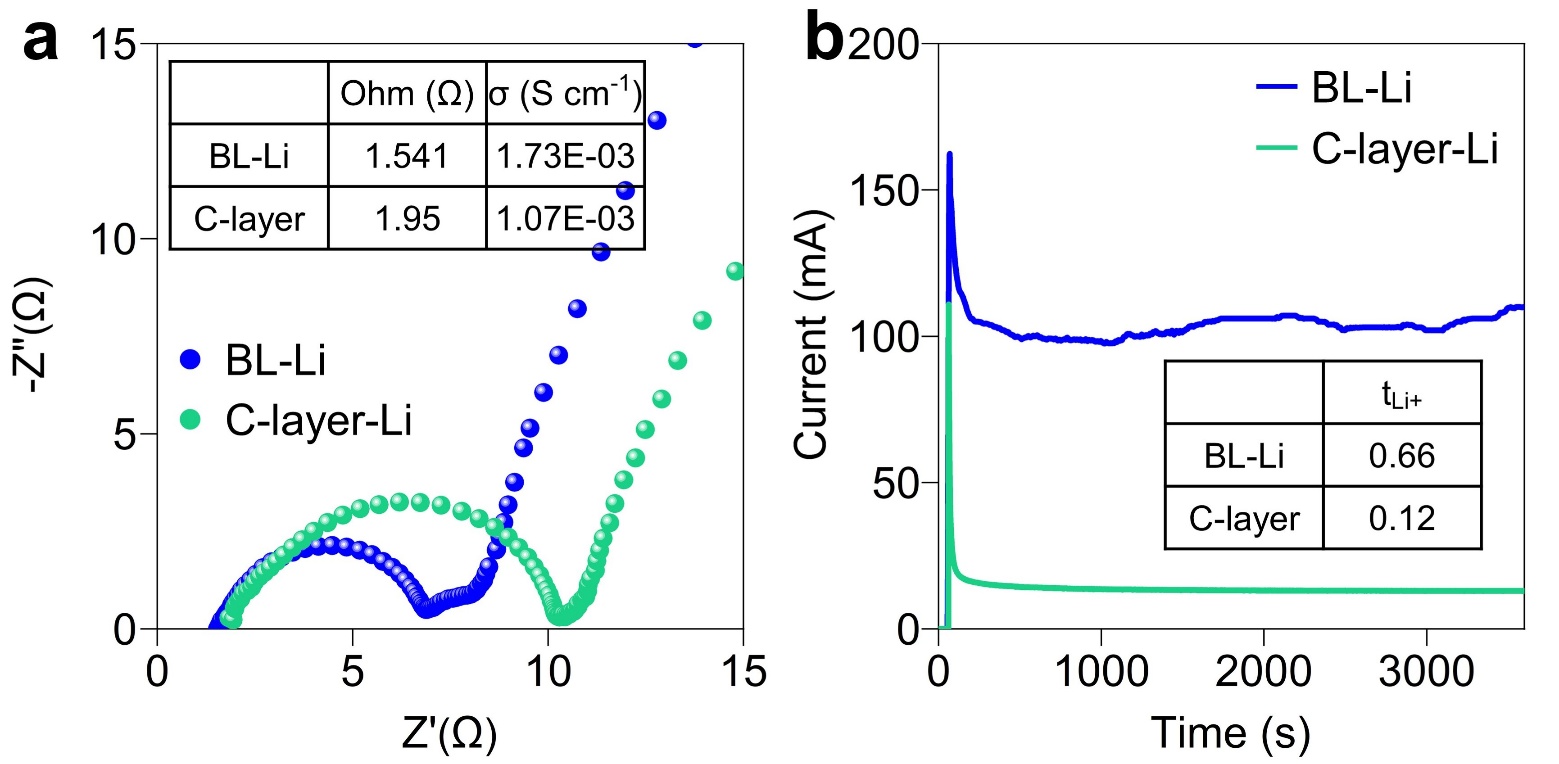
**

**Fig. S32** The Li^+^ ionic conductivity (**a**) and chronoamperometry profile and transference number (**b**) of BL-Li and C-layer-Li cells


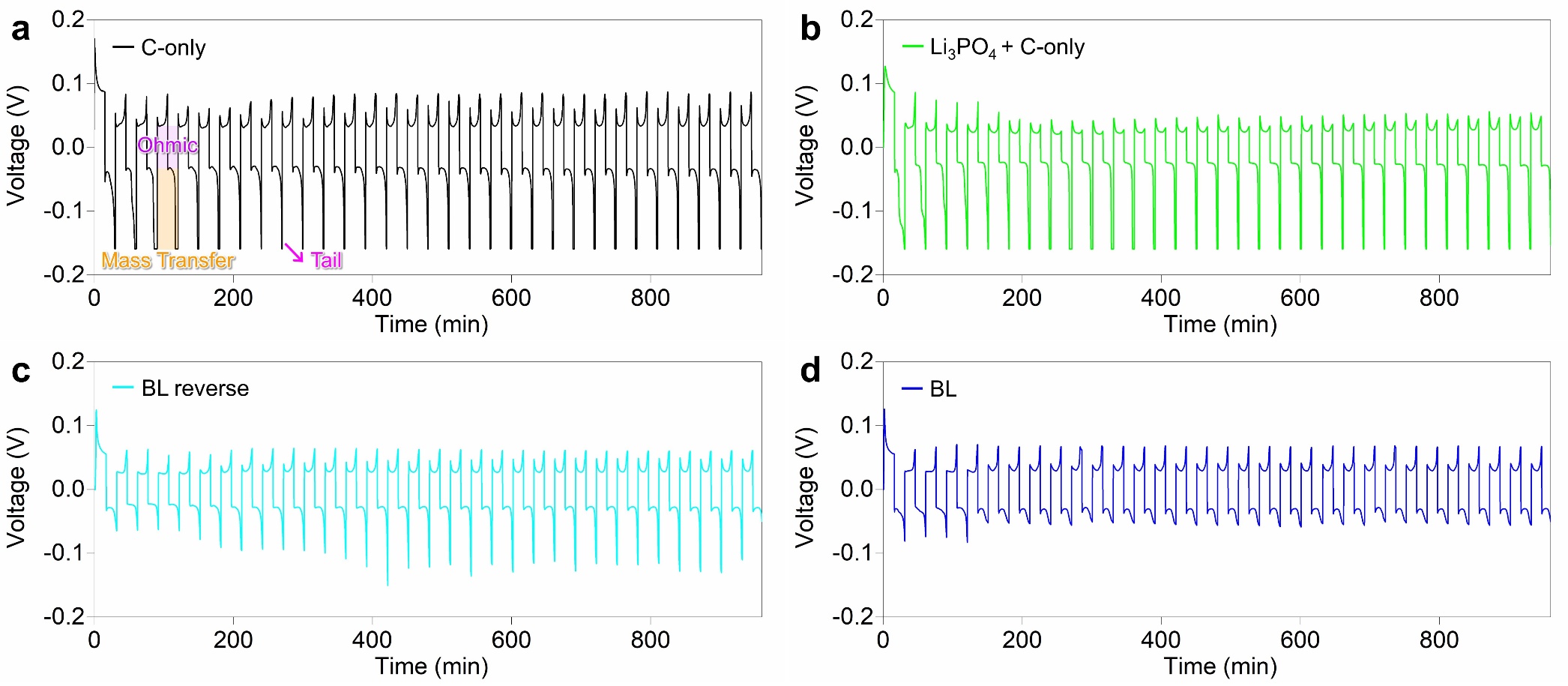


**Fig. S33** Voltage–time profiles of Li‖Li symmetric cells with different interfacial configurations at 4 mA cm^−2^ with a fixed capacity of 1 mAh cm^−2^ : (**a**) C-only, (**b**) Li₃PO₄ + C-only, (**c**) BL reverse, and (**d**) BL

**
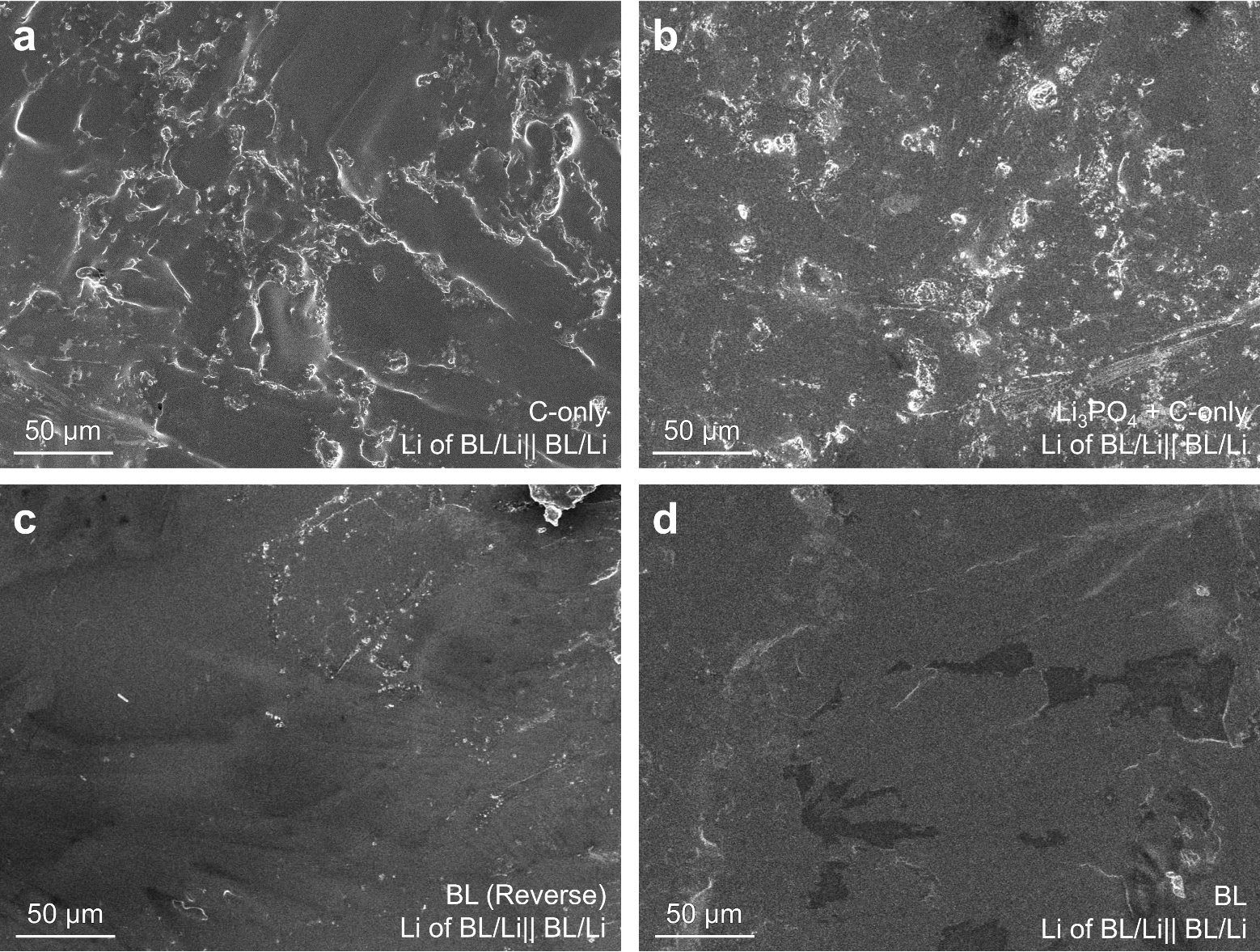
**

**Fig. S34** SEM images for Li after symmetric Li cells of (**a**) C-layer only, (**b**) Li_3_PO_4_ + C-only, (**c**) BL (reverse), and (**d**) BL at 4 mA cm^−2^ with a fixed capacity of 1 mAh cm^−2^ for 50 cycles


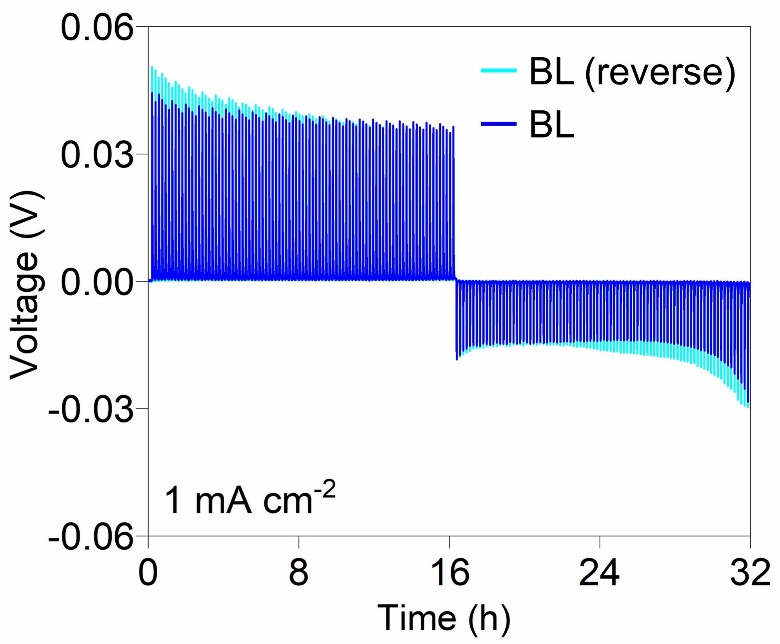


**Fig. S35** GITT profiles at 1 mA cm ^−2^ of BL (reverse)-Li and BL-Li cells


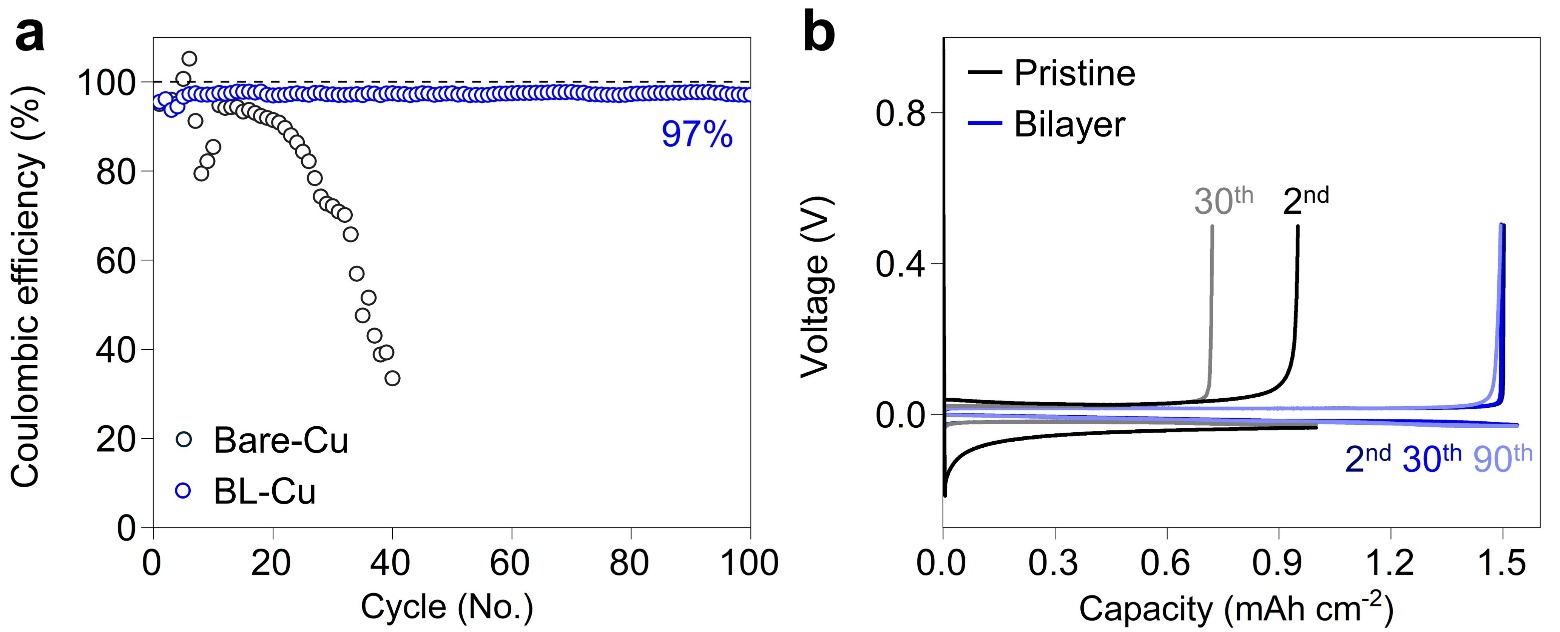


**Fig. S36** Comparison of the Coulombic efficiencies of Bare-Cu||Li and BL-Cu||Li cells at 1 mA cm^-2^ and 1 mAh cm^-2^ (**a**), and corresponding voltage profiles (**b**)

**
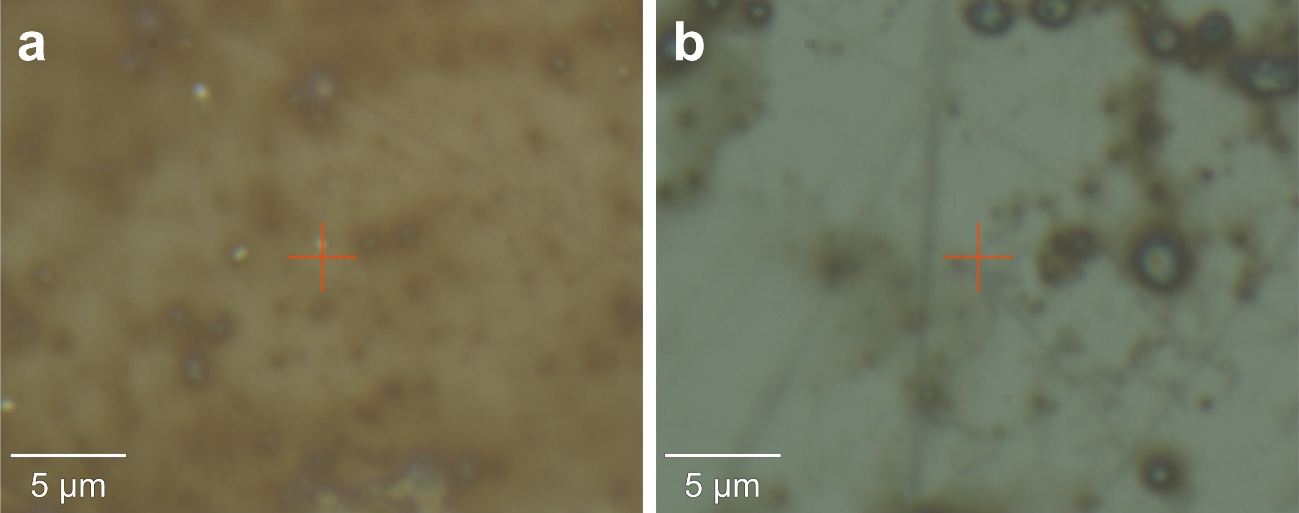
**

**Fig. S37** Optical images during In-situ Raman spectroscopy of Bare-Li (**a**) and BL-Li (**b**)

**
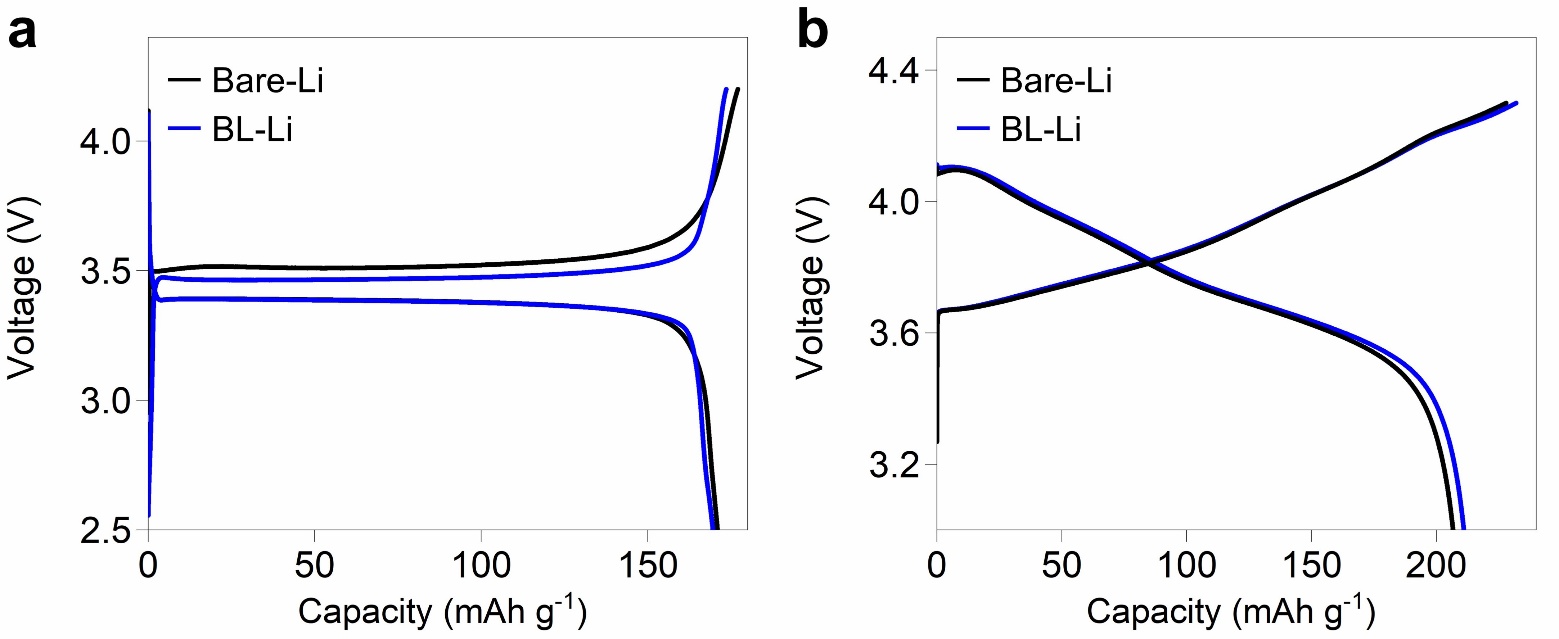
**

**Fig. S38** Galvanostatic charge–discharge curves of Li||LFP and Li||NCM811 at 0.2C


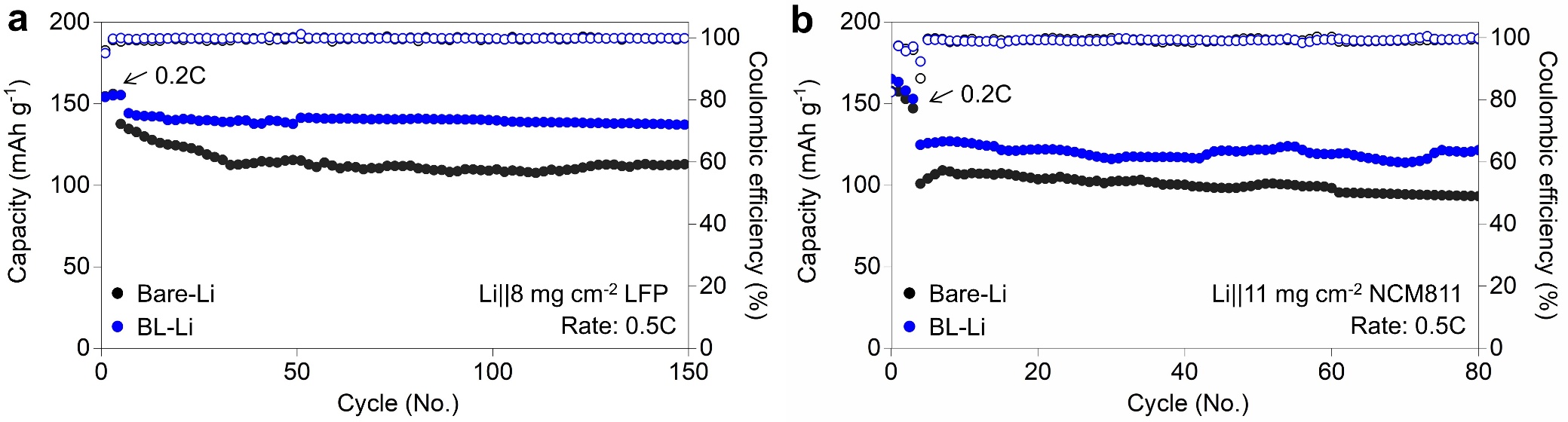


**Fig. S39** Cycling performance with higher loading cathodes of LFP and NCM811

**
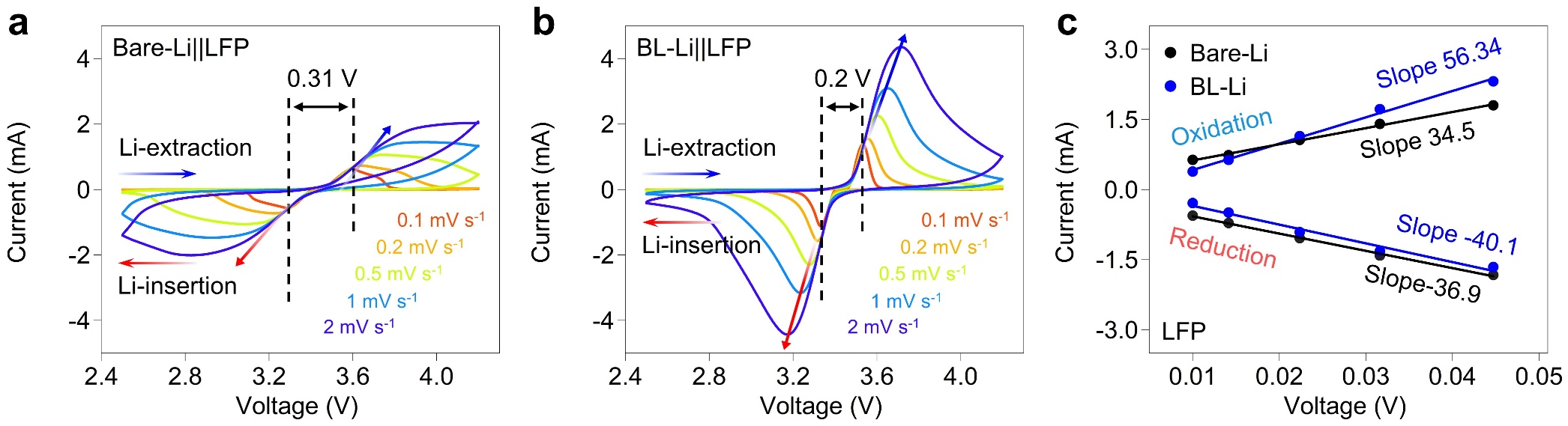
**

**Fig. S40** CV curves of Bare-Li‖LFP cell (**a**) and BL-Li‖LFP cell at different scan rates from 0.1 to 2 mV s^-1^ (**b**) and corresponding linear fitting results of oxidation/reduction peak currents (I_peak_) against the square root of the scan rates (ν^0.5^) (**c**)


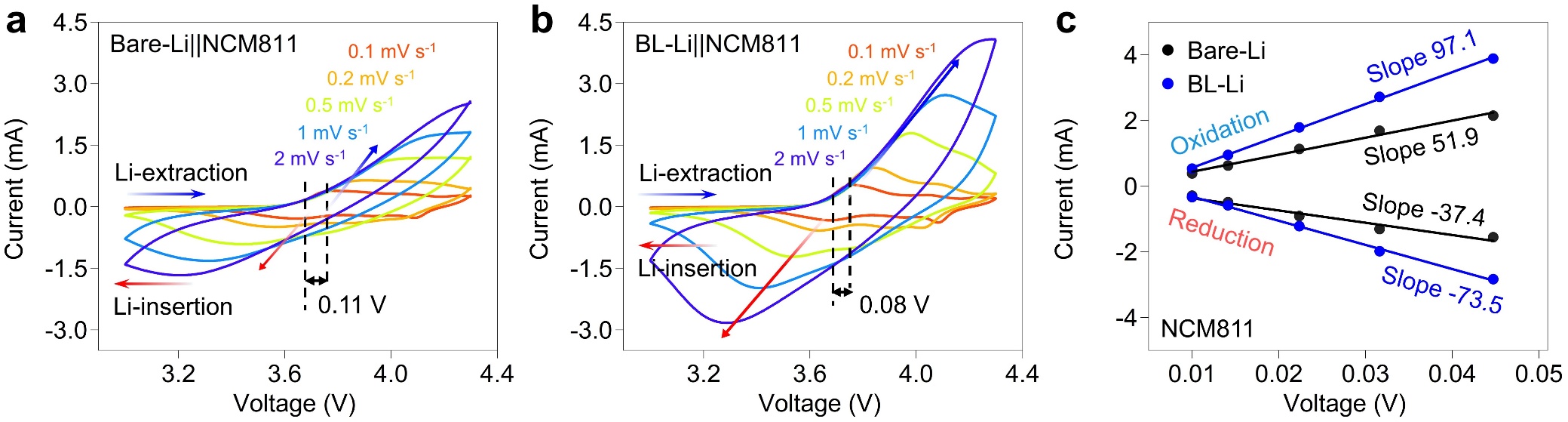


**Fig. S41** CV curves of Bare-Li‖NCM811 cell (**a**) and BL-Li‖NCM811 cell at different scan rates from 0.1 to 1 mV s^-1^ (**b**) and corresponding linear fitting results of oxidation/reduction peak currents (I_peak_) against the square root of the scan rates (ν^0.5^) (**c**)


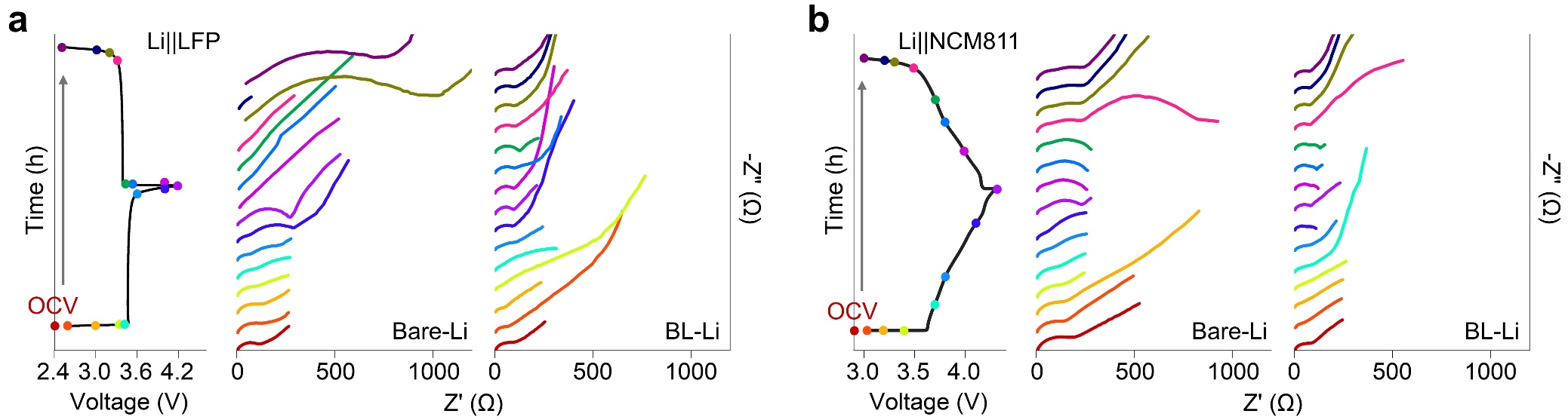


**Fig. S42** In-situ EIS results with LFP (**a**) and NCM811 (**b**) cycled in Bare-Li and BL-Li anodes during discharge/charge

**Table S1** Comparison of R_ohm_, R_sei,_ R_ct_ in the LFP cells with Bare-Li and BL-Li cells at various voltage stages

|  | **R_ohm_ (Ω)** | | **R_sei_ (Ω)** | | **R_ct_ (Ω)** | |
| --- | --- | --- | --- | --- | --- | --- |
|  | **Bare-Li** | **BL-Li** | **Bare-Li** | **BL-Li** | **Bare-Li** | **BL-Li** |
| **1** | 4.621 | 2.589 | 70.78 | 48.63 | 45.84 | 508.7 |
| **2** | 5.216 | 3.191 | 60.72 | 38.21 | 16.06 | 145.2 |
| **3** | 4.509 | 2.865 | 75.96 | 25.08 | 125.6 | 303 |
| **4** | 4.027 | 2.287 | 106.9 | 10.43 | 81.31 | 723.1 |
| **5** | 5.642 | 2.484 | 75.87 | 24.77 | 293.1 | 310.4 |
| **6** | 1.914 | 1.785 | 56.76 | 22.15 | 443 | 166.3 |
| **7** | 4.328 | 2.457 | 68.78 | 69.06 | 233.4 | 73.93 |
| **8** | 4.772 | 2.243 | 31.57 | 18.81 | 257.5 | 73 |
| **9** | 11.52 | 2.066 | 2305 | 99.96 | 1.108 | 32.89 |
| **10** | 11.59 | 2.138 | 337.5 | 38.18 | 14.86 | 86.23 |
| **11** | 10.36 | 2.259 | 318.8 | 12.54 | 48.83 | 69.24 |
| **12** | 9.848 | 2.108 | 332.4 | 36.23 | 18.97 | 60.29 |
| **13** | 32.06 | 2.089 | 533 | 43.77 | 776.1 | 47.84 |
| **14** | 10.64 | 2.106 | 335.4 | 37.15 | 23.18 | 47.76 |
| **15** | 15.4 | 2.086 | 2156 | 35.52 | 982.7 | 44.04 |

**Table S2** Comparison of R_ohm_, R_sei,_ R_ct_ in the 811 cells with Bare-Li and BL-Li cells at various voltage stages

|  | **R_ohm_ (Ω)** | | **R_sei_ (Ω)** | | **R_ct_ (Ω)** | |
| --- | --- | --- | --- | --- | --- | --- |
|  | **Bare-Li** | **BL-Li** | **Bare-Li** | **BL-Li** | **Bare-Li** | **BL-Li** |
| **1** | 6.604 | 3.149 | 108.8 | 14.95 | 58.45 | 96.4 |
| **2** | 4.23 | 3.069 | 109.1 | 42.5 | 266 | 78 |
| **3** | 4.01 | 2.273 | 142.1 | 18.99 | 187.1 | 1.076 |
| **4** | 4.534 | 2.221 | 46.17 | 36.4 | 45.02 | 263 |
| **5** | 3.671 | 2.474 | 113.6 | 36.75 | 402 | 217.8 |
| **6** | 2.107 | 2.202 | 42.21 | 24.84 | 767.4 | 4.724 |
| **7** | 2.96 | 2.745 | 43.27 | 31.51 | 682.2 | 5.716 |
| **8** | 4.099 | 2.664 | 54.96 | 75.24 | 182.4 | 2.485 |
| **9** | 4.076 | 1.273 | 31.79 | 58.06 | 219.4 | 2.571 |
| **10** | 3.814 | 2.712 | 55.75 | 61.26 | 209.2 | 2.1468 |
| **11** | 4.037 | 3.026 | 53.48 | 36.01 | 222.6 | 350 |
| **12** | 4.153 | 2.59 | 52.55 | 72.25 | 237.5 | 242.3 |
| **13** | 4.06 | 2.738 | 58.95 | 51.71 | 243.7 | 20.73 |
| **14** | 4.619 | 2.719 | 65.44 | 51.93 | 220.7 | 22.04 |
| **15** | 4.48 | 2.712 | 61.36 | 50.16 | 241.2 | 23.67 |

**
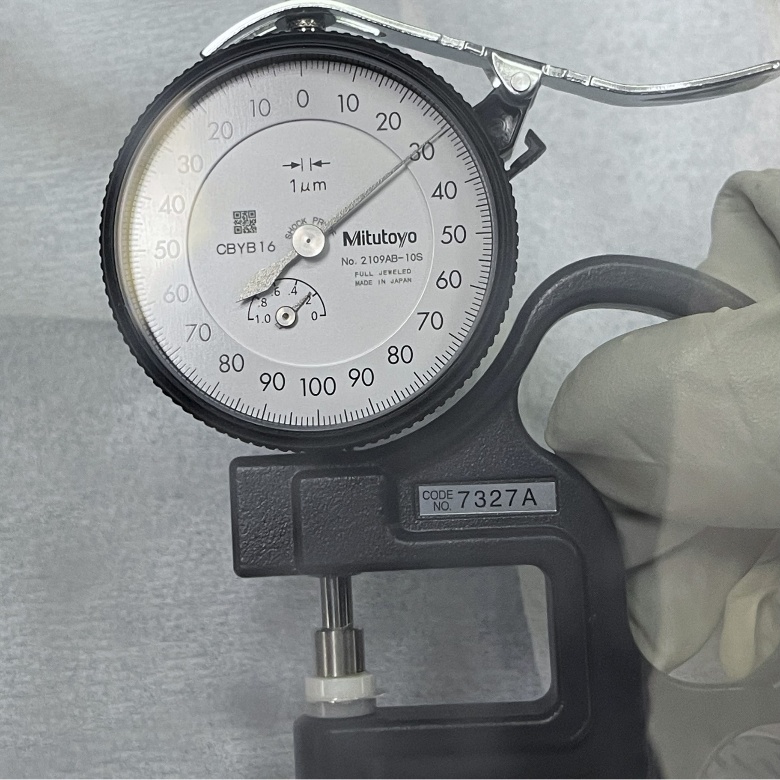
**

**Fig. S43** Photo image of thickness 30 μm lithium metal anode

**Table S3** Comparison of the electrochemical performance of modified metal lithium anodes paired with cathodes reported over the past three years in full batteries

|  | **Cathode** | **C-rate** | **Loading mass** | **Current density** | **Cycles** | **Refs.** |
| --- | --- | --- | --- | --- | --- | --- |
|  |  | C | mg cm^-2^ | mA cm^-2^ |  |  |
| **This work*** | LiFePO_4_ | 20 | 1.5 | 5.1 | 6000 | * |
| **1** |  | 1 | 1.2 | 0.204 | 400 | [S1] |
| **2** |  | 1 | 2.3 | 0.391 | 1200 | [S2] |
| **3** |  | 1 | 2.1 | 0.357 | 1500 | [S3] |
| **4** |  | 1 | 2 | 0.34 | 950 | [S4] |
| **5** |  | 1 | 4 | 0.68 | 1000 | [S5] |
| **6** |  | 1 | 11 | 1.87 | 1000 | [S6] |
| **7** |  | 1 | 2 | 0.34 | 1500 | [S7] |
| **8** |  | 1 | 3.5 | 0.595 | 600 | [S8] |
| **This work*** | LiNi_0.8_Co_0.1_Mn_0.1_O_2_ | 3 | 2.5 | 2.00625 | 500 | * |
| **9** |  | 3 | 1.2 | 0.963 | 1500 | [S1] |
| **10** |  | 1 | 1.8 | 0.4815 | 450 | [S9] |
| **11** |  | 0.5 | 3.5 | 0.468125 | 150 | [S10] |

**Supplementary References**

1. F. Tao, K. Yan, C. Dong, J. Wang, Q. Pan et al., Electric-dipole coupling ion-dipole engineering induced rational solvation-desolvation behavior for constructing stable solid-state lithium metal batteries. Angew. Chem. Int. Ed. **64**(21), e202503037 (2025). <https://doi.org/10.1002/anie.202503037>
2. T. Yang, X. Xu, S. Chen, Y. Yang, F. Li et al., A lithiophilic donor–acceptor polymer modified separator for high-performance lithium metal batteries. Angew. Chem. Int. Ed. **64**(9), e202420973 (2025). <https://doi.org/10.1002/anie.202420973>
3. H. Liu, F. Zhen, X. Yin, Y. Wu, K. Yu et al., Ultra-tough dynamic supramolecular ion-conducting elastomer induced uniform Li+ transport and stabilizes interphase ensures dendrite-free lithium metal anodes. Angew. Chem. Int. Ed. **64**(2), e202414599 (2025). <https://doi.org/10.1002/anie.202414599>
4. T. Naren, G.-C. Kuang, R. Jiang, P. Qing, H. Yang et al., Reactive polymer as artificial solid electrolyte interface for stable lithium metal batteries. Angew. Chem. Int. Ed. **62**(26), e202305287 (2023). <https://doi.org/10.1002/anie.202305287>
5. Y. Zhang, Y. Guo, K. Yong, Q. Wang, M. Yao et al., A large-capacity, superhigh-rate integrated lithium metal anode with top-down composition gradient enabled by polyantimonic acid. Energy Environ. Sci. **17**(16), 5819–5832 (2024). <https://doi.org/10.1039/d3ee04243j>
6. F. Zhen, H. Liu, Y. Wu, X. Zhou, W. Li et al., The spontaneous cascade optimization strategy of the double enrichment improves anion-derived solid electrolyte interphases to enable stable lithium-metal batteries. Energy Environ. Sci. **18**(10), 4690–4703 (2025). <https://doi.org/10.1039/d5ee01352d>
7. S. Xu, T. Naren, Y. Zhao, Q. Gu, T. Wai Lau et al., Soluble covalent organic frameworks as efficient lithiophilic modulator for high-performance lithium metal batteries. Angew. Chem. Int. Ed. **64**(15), e202422040 (2025). <https://doi.org/10.1002/anie.202422040>
8. L. Kong, Y. Li, C. Peng, Z. Zhao, J. Xiao et al., Achieving burst Li^+^ channels *via* quasi-two-dimensional fluorinated metal-organic framework modulating functionalized interface. Nat. Commun. **16**(1), 1885 (2025). <https://doi.org/10.1038/s41467-025-57106-z>
9. S. Duan, L. Zhang, Y. Zheng, Z. Li, Z. Liu et al., “Rigid exterior, soft interior” design enables high-voltage polyether electrolytes for quasi-solid-state batteries. Angew. Chem. Int. Ed. **64**(32), e202502728 (2025). <https://doi.org/10.1002/anie.202502728>
10. L. Kong, Y. Li, C. Peng, Z. Zhao, J. Xiao et al., Achieving burst Li^+^ channels *via* quasi-two-dimensional fluorinated metal-organic framework modulating functionalized interface. Nat. Commun. **16**(1), 1885 (2025). <https://doi.org/10.1038/s41467-025-57106-z>
